# Nonlinear Rayleigh wave propagation in a layered half-space in dual-phase-lag

**DOI:** 10.1038/s41598-023-29411-4

**Published:** 2023-02-07

**Authors:** A. A. Youssef, N. K. Amein, N. S. Abdelrahman, M. S. Abou-Dina, A. F. Ghaleb

**Affiliations:** 1 Department of Mathematics, Faculty of Science, Suez Canal University, Ismailia, Egypt; 2 Department of Mathematics, Faculty of Science, Cairo University, Giza, 12613 Egypt

**Keywords:** Engineering, Physics

## Abstract

We investigate nonlinear Rayleigh wave propagation in a layered thermoelastic medium composed of a slab rigidly bonded to the surface of a half-space under prescribed external thermal boundary conditions within the dual-phase-lag theory. The heat conduction coefficient for both the slab and the matrix have a linear dependence on temperature. Our aim is to assess the effect of temperature dependence of the heat conductivity, as well as the thermal relaxation times, on the process of wave propagation in the layered medium. Poincaré expansion of the solution in a small parameter and the generation of higher harmonics allow to evaluate the coefficient of this nonlinear coupling in the slab through heat wave propagation measurement. For the used numerical values, the results show that some characteristics of the problem, e.g. the temperature, heat flux and one stress component suffer jumps at the interface, while the other stress components are continuous there. The jump in the heat flux is noticeable only in the first order of nonlinearity. The existence of jumps at the interface may be of interest for measurements. Comparison with the case of the half-space showed that the presence of the slab contributes to faster damping of the solution with depth in the half-space.

## Introduction

There has been an increasing interest in the past few decades in the study of materials, especially those with complex structure, or metamaterials. Specific properties of such materials allow to manipulate elastic waves and to achieve particular goals, for example vibration attenuation or suppression, otherwise impossible to reach with natural materials. In particular, it is a problem of great significance to ensure the integrity of parts in the different devices that use such materials.

Examples of elastic metamaterials are the phononic crystals which have witnessed a rapid development in the past few decades. They are presently considered as an important component in science and technology due to their numerous applications as smart materials in intelligent microstructures in acoustic and vibration engineering, particularly in the field of acoustic filters and transducers, as well as advanced materials for noise control. Complex structures involving models of phononic crystals in linear or in nonlinear elastic media were explored in^1–5^ on the basis of nonlocal theories in order to put in evidence the existence of frequency band gaps, wave energy distribution among the frequencies, effect of elastic nonlinearity and initial stresses, and the diode non-reciprocal transnission which prohibits wave propagation in certain directions.

One of the most reliable ways to extract useful information is to use Rayleigh waves travelling at ultrasonic speeds in the bulk and at the surfaces of materials to detect flaws in the microstructure, assess the level of microscale damage and degradation, and evaluate different material parameters. Nonlinear Rayleigh waves and higher order harmonic generation can be efficiently applied to study the nonlinearities occuring in the medium due to different couplings and the dependence of the various material parameters on strain, temperature and other factors as well. Generally, the nonlinearity parameters have higher sensitivity than linear ultrasonic parameters to changes in the microstructure. Different applications of nonlinear Rayleigh wave propagation in elastic media may be found in^6–9^.

In many cases, bulk metamaterial substrates are covered by thin layers of a different material. Such layered material may have interesting features and various applications, e.g. signal processing, construction engineering, energy harvesting and other (c.f.^10–12^).

The study of wave propagation in media with microstucture is best carried out within the theory of extended thermodynamics. In fact, the latter provides a richer set of thermodynamical variables compared to classical thermodynamics. Heat flux is now independent of temperature and has its own evolution equation which replaces the classical Fourier law for heat conduction. In dual-phase-lag (DPL), there are two thermal relaxation times, one for each of the heat flux and temperature, which may account for different relaxational and dissipative phenomena in the thermoelastic medium. Extensive work exists on wave propagation in solids of various geometries in extended thermodynamics. Only a few are cited here for reference. Ramadan^13^ proposed a semi-analytical solution for linear transient heat transfer in multi-layered thermoelastic media within the framework of dual-phase-lag. Askarizadeh and Ahmadikia^14^ solved a linear problem of heat transfer in a thermoelasstic slab with periodic surface heat flux with DPL. Sur and Kanoria^15^ investigated thermoelastic interactions in a three-dimensional homogeneous and isotropic sandwich structure in DPL under time-dependent thermal load within the linear theory. Ahmed and Abou-Dina^16^ studied linear wave propagation in a piezo-thermoelastic slab within DPL. Ai et al.^17^ analyzed the linear thermo-mechanical problem for multi-layered media in extended thermodynamics based on Lord-Shulman model. Ahmed et al.^18,19^ considered a linear, two-dimensional initial-boundary-value problem of heat wave propagation in a thick slab of anisotropic thermal conductor within the dual-phase-lag model. Lately, Youssef et al.^20^ investigated the nonlinear Rayleigh wave propagation in a half-space of a thermoelastic material within dual-phase-lag, with temperature dependent thermal conductivity using Poincaré small parameter expansion.

The present work is an extension of^20^ to the case of a layered thermoelastic medium consisting of a thick layer rigidly bonded to a half-space, with temperature dependent thermal conductivity for both. The main difference between the present work and the above reference consists of considering a layered medium with an interface, instead of a single half-space. In both cases, the considered system of equations is linear, except for a single nonlinearity in the evolution law for heat flux, pertaining to the temperature dependence of heat conductivity. Other potential nonlinear mechanical or thermomechanical couplings were disregarded for the sake of brevity. The nonlinear Rayleigh wave propagation here may be used to test the response of the layer, when the half-space (the matrix) is a material with known properties. The applied boundary conditions at the interface are those commonly derived from the field equations by well-known procedures of general Continuum Mechanics. The solution is expressed as a Poincaré expansion in a small parameter and only the first two orders of approximation are retained for the present purposes. The generation of higher harmonics allows to evaluate the coefficient of the nonlinear coupling in the slab through heat wave propagation measurement. The obtained particular solution allows to explain the main features under consideration. However, when the material has a different structure, for example nonlinear elastic bodies or phononic crystals, and when the phenomenon under investigation is different than that in the present work, then other solutions where the traditional small expansion method is no more valid can be obtained by various existing methods. For the considered case study, the results show that the temperature and one stress component along the direction of wave propagation suffer jumps at the interface, while the other stress components are continuous there. The heat flux components are continuous at the interface at the first approximation, but appear to have jumps there at the second approximation. The existence of jumps at the interface may be of interest for measurements. Comparison has been carried out with the case of a half-space recently published by the authors. In particular, it is shown that the presence of a thick slab bonded to the half-space produces faster damping with depth of all the functions in the latter.

## Problem formulation

**Figure 1:**
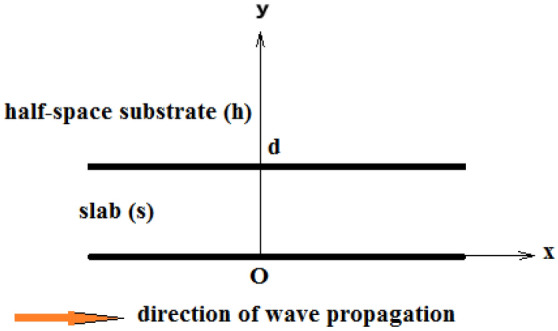
Geometry of the problem.

We consider nonlinear Rayleigh wave propagation in a layered half-space of transversely isotropic thermoelastic materials composed of a slab rigidly bonded to a half-space substrate. The problem is described in a system of orthogonal Cartesian coordinates (*x*, *y*, *z*) with origin *O* placed on the boundary of the half-space, the *y*-coordinate being directed into the depth of the material, as shown in Fig. 1. Moreover, there is no dependence of the solution on *z*-coordinate.

## Basic equations

In the following, $u_x,u_y$ denote the mechanical displacement components, $v_x,v_y$- the corresponding velocity components, $\sigma _{xx}, \sigma _{yy}, \sigma _{xy}$—the in-plane stress components, $\theta$—the temperature as measured from a reference temperature $\theta _0$ and $q_x, q_y$- the heat flux components. All other stress components vanish identically.

Body forces and heat sources are disregarded. The dimensionless equations of plane thermoelasticity for a transversely isotropic material within the theory of extended thermodynamics to be considered are expressed in^19,20^. They involve the equations of motion, the equation of energy, the evolution laws for the heat flux components and the generalized Hooke’s laws differentiated w.r.t. time. More details may be found in these two references. The characteristic quantities for temperature, length, time and heat flux used in the dimension analysis are respectively given by:

$$\begin{aligned} \Theta _{0}=\theta _{0}, \quad L_{0}= \sqrt{\frac{\tau _0 K_0}{\rho C_e}}, \quad T_0 = \tau _0, \quad Q_{0}=\Theta _{0} \sqrt{\frac{\rho C_e K_0 }{\tau _0 }}, \end{aligned}$$

by which it is seen that the characteristic velocity

$$\begin{aligned} \frac{L_0}{T_0} = \sqrt{\frac{K_0}{\rho C_e}} \, \frac{1}{\sqrt{\tau _0}} \end{aligned}$$

is closely related to the velocity of second sound. Here, $\rho$ denotes the mass density, $K_0$—a characteristic heat conductivity, $C_e$ —the specific heat and $\tau _0$—a characteristic thermal relaxation time.

The system of governing equations to be considered here below in dimensionless form is given in^20^. It is formulated within the theory of extended thermodynamics and involves eight first-order partial differential equations: Two equations of motion, the equation of energy, two evolution laws for the heat flux components and three constitutive relations for the identically non-vanishing stress components (these are differentiated w.r.t. time). The relaxation times have been taken the same for both spatial directions for simplicity. Eight unknown functions are involved in this system of equations: Two velocity components, temperature, two components of heat flux and three stress components. The mechanical displacement components may then be determined by quadrature after the eight basic unknown functions have been obtained. Details may be found in the above reference.

The system of equations reads:

1 $$\begin{aligned}{} & {} \frac{\partial v_x}{\partial t}-\beta _1\left( \frac{\partial \sigma _{xx}}{\partial x}-\beta _4\frac{\partial \theta }{\partial x}+\frac{\partial \sigma _{xy}}{\partial y}\right) =0, \end{aligned}$$

2 $$\begin{aligned}{} & {} {\frac{\partial v_y}{\partial t}-\beta _1\left( \frac{\partial \sigma _{xy}}{\partial x}-\beta _4\frac{\partial \theta }{\partial y}+\frac{\partial \sigma _{yy}}{\partial y}\right) =0,} \end{aligned}$$

3 $$\begin{aligned}{} & {} {\frac{\partial \theta }{\partial t}+\beta _2\left( \frac{\partial v_x}{\partial x}+\frac{\partial v_y}{\partial y}\right) +\left( \frac{\partial \, q_x}{\partial x}+\frac{\partial \, q_y}{\partial y}\right) =0,} \end{aligned}$$

4 $$\begin{aligned}{} & {} { \tau _q\frac{\partial q_x}{\partial t}+q_x+K_{11}(\theta ) \left( \frac{\partial \theta }{\partial x}+ \tau _{\theta } \frac{\partial ^2\theta }{\partial x\partial t}\right) =0,} \end{aligned}$$

5 $$\begin{aligned}{} & {} { \tau _q\frac{\partial q_y}{\partial t}+q_y+K_{22}(\theta )\left( \frac{\partial \theta }{\partial y}+ \tau _{\theta } \frac{\partial ^2\theta }{\partial y\partial t}\right) =0,} \end{aligned}$$

6 $$\begin{aligned}{} & {} {\frac{\partial }{\partial t}\left( \beta \sigma _{xx}-\alpha \sigma _{yy}\right) -\frac{\partial v_x}{\partial x}=0,} \end{aligned}$$

7 $$\begin{aligned}{} & {} {\frac{\partial }{\partial t}\left( -\alpha \sigma _{xx}+\beta \sigma _{yy}\right) -\frac{\partial v_y}{\partial y}=0,} \end{aligned}$$

8 $$\begin{aligned}{} & {} {\frac{\partial \sigma _{xy}}{\partial t}-\frac{\partial v_x}{\partial y}-\frac{\partial v_y}{\partial x}=0,} \end{aligned}$$

with

$$\begin{aligned} \beta _1 = \frac{\mu \tau _0 C_e}{K_0}, \quad \beta _2 = \frac{\gamma }{\rho C_e}, \quad \beta _3 = \frac{\lambda }{\mu }, \quad \beta _4 = \frac{\gamma \theta _0}{\mu }, \end{aligned}$$

and

$$\begin{aligned} \alpha = \frac{\lambda }{4 ( \lambda + \mu )}, \qquad \beta = \frac{\lambda + 2 \mu }{4 ( \lambda + \mu )}. \end{aligned}$$

Here, $\rho$ is the mass density, $\lambda , \mu$- Lam$\acute{e}$ coefficients, $\gamma$- the thermoelastic coefficient, $K_{11}, K_{22}$- the coefficients of heat conduction and $\tau _q, \tau _{\theta }$- the relaxation times related to temperature and heat flux respectively. Nonlinearity appears only in the evolution equations for heat flux Eqs. (4) and (5) in connection with the dependence on temperature of the heat conduction coefficients.

### Particular solution

The propagation of nonlinear waves has attracted a great deal of attention in the past few years because of many important applications. Different theoretical approaches have been proposed to tackle problems of nonlinear elastic wave propagation in complex structured media, for example granular materials and phononic crystals. One may refer here to the incremental harmonic balance method^2^ that is an efficient replacement of the traditional perturbation methods for treating problems involving materials with strongly nonlinear periodic structures. Such techniques are basically related to nonlocal theories of Continuum Mechanics and are expected to explain a broader spectrum of phenomena.

For the present purposes, when nonlinearity is of the type explained above and shown in the evolution equations for heat flux, it is sufficient to look for a particular solution for all the unknowns of the problem to describe Rayleigh wave propagation, for which the amplitude decreases exponentially in depth into the medium, in the form of usual Poincaré expansions in a small parameter $\varepsilon$, say. The effect of the first order of nonlinearity will appear separately in the presented figures. Subsequent orders of approximation will add much smaller terms to the solution, and therefore need to be taken in consideration only when the need for increasing precision arises. In what follows, only the frst two orders of approximation will be considered. The approximate solution takes the form:

9 $$\begin{aligned}{} & {} \{ v_x, v_y, \theta , \sigma _{xx}, \sigma _{yy}, \sigma _{xy}, q_x, q_y \}(x,y,t) = \nonumber \\{} & {} \varepsilon \{ v^*_x, v^*_y, \theta ^*, \sigma ^*_{xx}, \sigma ^*_{yy}, \sigma ^*_{xy}, q^*_x, q^*_y \}(y) \, e^{i(k x-\omega _i t) - \omega _r t} + \nonumber \\+ & {} \varepsilon ^2 \{ v^{**}_x, v^{**}_y, \theta ^{**}, \sigma ^{**}_{xx}, \sigma ^{**}_{yy}, \sigma ^{**}_{xy}, q^{**}_x, q^{**}_y \}(y) \, e^{2i(k x - \omega _i t) -2 \omega _r t} + \ldots , \end{aligned}$$

where the “starred”  and the “double-starred”  quantities denote the amplitudes of the corresponding functions at the first and the second approximations respectively, and $\varepsilon$ is an adequately chosen positive small parameter representing the amplitude of variation of the temperature as measured from the reference temperature $\Theta _0$. Here, *k* denotes the wavenumber, $\omega _r$- the frequency and $\omega _i$- the time damping, or attenuation coefficient. The dependence of some of the unknowns on the others is taken into account in the forthcoming relations. As the only considered nonlinearity arises from a linear dependence of the heat conductivity on temperature, it is sufficient to limit the small parameter expansions of the unknown functions to the first two orders of approximation as noted earlier.

In the present work, the normal mechanical load and the temperature have prescribed values at the boundaries. In order to concentrate our attention on the nonlinearity of the equations, we simply find particular solutions for the linear approximation for selected wave number, frequency and attenuationg coefficient, rather than analyzing in detail the arising dispersion relation. The influence on surface wave propagation of the linear dependence of the heat conduction coefficient on temperature, as well as thermal relaxation times, is investigated.

Substituting from Eq. (9) into the basic Eqs. (1)–(8) and denoting $D= \frac{d}{dy}$, one is left with a system of homogeneous linear ordinary differential equations of the first order, and a system of non-homogeneous linear ordinary differential equations of the first order, in the first two orders of approximation respectively. In this procedure, we have set

10 $$\begin{aligned} K_{11} \left( \theta \right) =K_{22} \left( \theta \right) =K_{0} (1+\eta \, \theta ), \end{aligned}$$

thus allowing for a linear dependence of the heat conduction coefficient on temperature in the half-space. For the slab, a similar dependence on temperature is assumed, with $K_0$ replaced by $K_s$. Such a dependence may be relevant, especially at high temperatures when the material characteristics are no longer constants. We assume that parameter $\eta$ has order of magnitude equal to unity.

### Wave amplitude and parameter $\eta$

From (10) it follows that

11 $$\begin{aligned} \frac{d K_{11}}{d \theta } = \frac{d K_{22}}{d \theta } = K_0 \eta , \end{aligned}$$

so that parameter $\eta$ only serves to modulate the value $K_0$, and may be determined experimentally from the slope of the curve for function $K_{11}(\theta )$. As to the small parameter $\varepsilon$, it is taken as a measure of the amplitude of the propagating linear waves. The wave amplitude of the nonlinear component is $\varepsilon ^2$, and its contribution is therefore expected to be much smaller than that of the linear wave. The effect of the nonlinear wave on the behavior of the solution, and more precisely on the heat flux, will appear subsequently while discussing the numerical results.

### Governing equations at the first two orders of approximation

The system of homogeneous linear differential equations of the first order:

12 $$\begin{aligned} D \textit{v}^*_x= & {} A_1 \, v ^*_y +A_2 \, \sigma _{xy}^*, \end{aligned}$$

13 $$\begin{aligned} D v ^*_y= & {} A_{3} \, v ^*_x +A_4 \, \sigma ^*_{yy},\end{aligned}$$

14 $$\begin{aligned} D \theta ^*= & {} A_5 \, q^*_y, \end{aligned}$$

15 $$\begin{aligned} D \sigma ^*_{yy}= & {} A_{6} \, v ^*_y +A_1 \, \sigma ^*_{xy} +A_7 \, q^*_y, \end{aligned}$$

16 $$\begin{aligned} D \sigma ^*_{xy}= & {} A_8 \, v ^*_x + A_9 \, \theta ^*+A_3\sigma ^*_{yy}, \end{aligned}$$

17 $$\begin{aligned} D q^*_y= & {} A_{10} \, \theta ^*+A_{11} \, v ^*_x +A_{12} \, \sigma ^*_{yy} , \end{aligned}$$

together with

18 $$\begin{aligned} \sigma ^*_{xx}= & {} A_{13} \, v ^*_x +A_{14 } \, \sigma ^*_{yy}, \end{aligned}$$

19 $$\begin{aligned} q^*_x= & {} A_{15 } \, \theta ^* . \end{aligned}$$

The system of non-homogeneous linear differential equations of the first order:

20 $$\begin{aligned} D v ^{**}_x= & {} A_{16} \, v ^{**}_y +A_{17} \, \sigma ^{**}_{xy}, \end{aligned}$$

21 $$\begin{aligned} D v ^{**}_y= & {} A_{18} \, v ^{**}_x + A_{19} \, \sigma ^{**}_{yy}, \end{aligned}$$

22 $$\begin{aligned} D \theta ^{**}= & {} A_{20} \, q^{**}_y +A_{31} \, \theta ^* D \theta ^*, \end{aligned}$$

23 $$\begin{aligned} D \sigma ^{**}_{yy}= & {} A_{21} \, v ^{**}_y +A_{16} \, \sigma ^{**}_{xy} +A_{22}q^{**}_y +A_{32} \theta ^* D \theta ^*, \end{aligned}$$

24 $$\begin{aligned} D \sigma ^{**}_{xy}= & {} A_{23} \, v ^{**}_x +A_{24} \, \theta ^{**}+A_{18} \, \sigma ^{**}_{yy}, \end{aligned}$$

25 $$\begin{aligned} D q^{**}_y= & {} A_{25 } \, \theta ^{**}+A_{26} \, v ^{**}_x +A_{27} \, \sigma ^{**}_{yy} + A_{33} \, \theta ^{*2} , \end{aligned}$$

together with

26 $$\begin{aligned} \sigma ^{**}_{xx}= & {} A_{28}{} \textit{v}^{**}_x +A_{29}\sigma ^{**}_{yy}, \end{aligned}$$

27 $$\begin{aligned} q^{**}_x= & {} A_{30}\theta ^{**}+A_{34} \theta ^{*2}. \end{aligned}$$

and $A_{j},j=1,2,...,34$ are constants listed in Supplementary Appendix A. It clearly appears that quadratic expressions in $\theta ^*$ will be responsible for the generation of the solution at the second order of approximation.

## Solution of the problem

### The homogeneous system

#### Half-space

28 $$\begin{aligned} {\left( \begin{array}{l} D v_x^* \\ D v_y^* \\ D \theta ^* \\ D \sigma _{yy}^* \\ D \sigma _{xy}^* \\ D q_y^* \\ \end{array} \right) =\left( \begin{array}{llllll} 0 &{} A_1 &{} 0 &{} 0 &{} A_2 &{} 0 \\ A_3 &{} 0 &{} 0 &{} A_4 &{} 0 &{} 0 \\ 0 &{} 0 &{} 0 &{} 0 &{} 0 &{} A_5 \\ 0 &{} A_6 &{} 0 &{} 0 &{} A_1 &{} A_7 \\ A_8 &{} 0 &{} A_9 &{} A_3 &{} 0 &{} 0 \\ A_{10} &{} 0 &{} A_{11} &{} A_{12} &{} 0 &{} 0 \\ \end{array} \right) \left( \begin{array}{l} v_x^* \\ v_y^* \\ \theta ^* \\ \sigma _{yy}^* \\ \sigma _{xy}^* \\ q_y^* \\ \end{array} \right) .} \end{aligned}$$

Assuming a solution of the form $e^{\xi _h y}$, the characteristic equation for the eigenvalues $\xi _h$ for this system of equations is obtained as:

29 $$\begin{aligned} \xi ^{6} _h- A \xi ^{4}_h + B \xi ^{2}_h - C = 0, \end{aligned}$$

where *A*, *B* and *C* are constants listed in Supplementary Appendix A.

Only three roots of this equation, $\xi _{nh}, n=1,2,3$ with positive real parts, will contribute to the bounded solution. Following Eq. (29), any imaginary part of $\xi _h$ will result in a circular function of sine or cosine in the solution, i.e. an amplitude that is oscillating in y while damped exponentially in y. In the end, only the real part of the solution will have physical meaning. The solution of the system of equations (28) may be written conveniently in the form:

30 $$\begin{aligned}{} & {} {v_x^*=\sum _{n=1}^3 \text { }v_{1nh} \,M_{nh } e^{-\xi _{nh} y},} \end{aligned}$$

31 $$\begin{aligned}{} & {} {v_y^*=\sum _{n=1}^3 v_{2 n} \, M_{nh} e^{-\xi _{nh}y},}\end{aligned}$$

32 $$\begin{aligned}{} & {} {\theta ^*=\sum _{n=1}^3 v_{3nh} \, M_{nh} e^{-\xi _{nh} y},}\end{aligned}$$

33 $$\begin{aligned}{} & {} {\sigma _{yy}^*=\sum _{n=1}^3 v_{4nh} \, M_{nh} e^{-\xi _{nh}y},}\end{aligned}$$

34 $$\begin{aligned}{} & {} {\sigma _{xy}^*=\sum _{n=1}^3 v_{5nh} \, M_{nh} e^{-\xi _{nh}y},}\end{aligned}$$

35 $$\begin{aligned}{} & {} {q_y^*=\sum _{n=1}^3 v_{6nh} \, M_{nh}e^{-\xi _{nh}y}.} \end{aligned}$$

where $v_{mnh}, m=1,2,...,6, \, n=1,2,3$ are constants listed in Supplementary Appendix B. The remaining two solution functions may now be calculated from Eqs. (18) and (19).

#### Slab

The solution of the system of equations (28) may be written conveniently in the form:

36 $$\begin{aligned}{} & {} {v_x^*=\sum _{n=1}^3 v_{1ns} \,M_{n s} e^{-\xi _{n s} y}+\sum _{n=1}^3 \text { }V_{1ns} \,m_{n s} e^{\xi _{n s} y},}\end{aligned}$$

37 $$\begin{aligned}{} & {} {v_y^*=\sum _{n=1}^3 v_{2 n s} \, M_{n s } e^{-\xi _{n s}y}+\sum _{n=1}^3 \text { }V_{2ns} \,m_{n s} e^{\xi _{n s} y},} \end{aligned}$$

38 $$\begin{aligned}{} & {} {\theta ^*=\sum _{n=1}^3 v_{3 n s} \, M_{n s } e^{-\xi _{n s}y}+\sum _{n=1}^3 \text { }V_{3ns} \,m_{n s} e^{\xi _{n s} y},}\end{aligned}$$

39 $$\begin{aligned}{} & {} {\sigma _{yy}^*=\sum _{n=1}^3 v_{4n s} \, M_{n s} e^{-\xi _{n s}y}+\sum _{n=1}^3 \text { }V_{4ns} \,m_{n s} e^{\xi _{n s} y},}\end{aligned}$$

40 $$\begin{aligned}{} & {} {\sigma _{xy}^*=\sum _{n=1}^3 v_{5 n s} \, M_{n s} e^{-\xi _{n s}y}+\sum _{n=1}^3 \text { }V_{5ns} \,m_{n s} e^{\xi _{n s} y},}\end{aligned}$$

41 $$\begin{aligned}{} & {} {q_y^*=\sum _{n=1}^3 v_{6n s} \, M_{n s}e^{-\xi _{n s}y}+\sum _{n=1}^3 \text { }V_{6ns} \,m_{n s} e^{\xi _{n s} y}.} \end{aligned}$$

where $v_{in s}$ and $V_{in s}, i=1,2,...,6, \, n=1,2,3$ are constants listed in Supplementary Appendix C. The remaining two solution functions may now be calculated as explained above.

#### Boundary conditions for the homogeneous system

There exists in the literature a multitude of boundary conditions for similar problems to the one considered here (see Ramadan^13^). For the present case, there are 9 boundary conditions, six of them at the interface between the slab and the matrix, and three at the outer boundary of the medium:

Boundary conditions at the interface $y=d$: these can be obtained from the field equations by the usual formalism of Continuum Mechanics, under the general title of “jump conditions”. Additionally, one uses the perfect bonding conditions between the layers. Denoting by subscripts “s”  and “d”  the quantities belonging to the slab and to the half-space respectively, one has:

42a $$\begin{aligned} v_{xs}&= v_{xh}, \end{aligned}$$

42b $$\begin{aligned} v_{ys}&= v_{yh}, \end{aligned}$$

42c $$\begin{aligned} \sigma _{xys}&= \sigma _{xyh}, \end{aligned}$$

42d $$\begin{aligned} \sigma _{yys}&= \sigma _{yyh}, \end{aligned}$$

42e $$\begin{aligned} K_{s} \, \theta _{s}&= K_{0} \, \theta _{h} \end{aligned}$$

42f $$\begin{aligned} K_{s} \, \theta _{,ys}&= K_{0} \, \theta _{,yh} \end{aligned}$$

Boundary conditions at the external boundary of the slab $y=0$:

43a $$\begin{aligned} \sigma _{xys}&= f_1^{*}, \end{aligned}$$

43b $$\begin{aligned} \sigma _{yys}&= f_2^{*}, \end{aligned}$$

43c $$\begin{aligned} \theta _{s}&= f_3^{*}. \end{aligned}$$

It is seen at once that the temperature has a jump at the interface. This is a direct consequence of the Cattaneo evolution equation for the heat flux. It takes the particular form shown in Eq. (43d) for the present geometrical configuration. The value of this jump is:

43d $$\begin{aligned}{}[ \theta ] \equiv \theta _h(d) - \theta _s(d) = \frac{K_{s} - K_{3h}}{K_{0}} \, \theta _s(d). \end{aligned}$$

Applying the boundary conditions to determine the $M_{ih}$, $M_{is}$ and $m_{is}$ one gets the system of equations given in Supplementary Appendix H. This system is cast in the following matricial form

44 $$\begin{aligned} \begin{pmatrix} M_{1s} \\ M_{2s} \\ M_{3s} \\ m_{1s} \\ m_{2s} \\ m_{3s} \\ M_{1h} \\ M_{ 2h} \\ M_{3h} \\ \end{pmatrix}=\begin{pmatrix} \Lambda _1 &{} \Lambda _2 &{} \Lambda _3 \\ \Lambda _4&{} \Lambda _5 &{} \Lambda _6\\ \Lambda _7&{}\Lambda _8 &{} \Lambda _9 \\ \end{pmatrix}^{-1}\begin{pmatrix} 0\\ 0\\ 0\\ 0\\ 0 \\ 0 \\ f_1^\star \\ f_2^ \star \\ f_3^\star \\ \end{pmatrix}. \end{aligned}$$

where $\Lambda _{1}-\Lambda _{9}$ are matrices given in Supplementary Appendix G.

### The non-homogeneous system

#### Half-space

$$\begin{aligned} {\left( \begin{array}{l} D v_x^{\text {**}} \\ D v_y^{\text {**}} \\ D \theta ^{\text {**}} \\ D \sigma _{yy}^{\text {**}} \\ D \sigma _{xy}^{\text {**}} \\ D q_y^{\text {**}} \\ \end{array} \right) =\left( \begin{array}{llllll} 0 &{} A_{16} &{} 0 &{} 0 &{} A_{17} &{} 0 \\ A_{18} &{} 0 &{} 0 &{} A_{19} &{} 0 &{} 0 \\ 0 &{} 0 &{} 0 &{} 0 &{} 0 &{} A_{20} \\ 0 &{} A_{21} &{} 0 &{} 0 &{} A_{16} &{} A_{22} \\ A_{23} &{} 0 &{} A_{24} &{} A_{18} &{} 0 &{} 0 \\ A_{25} &{} 0 &{} A_{26} &{} A_{27} &{} 0 &{} 0\text { } \\ \end{array} \right) \left( \begin{array}{l} v_x^{\text {**}} \\ v_y^{\text {**}} \\ \theta ^{\text {**}} \\ \sigma _{yy}^{\text {**}} \\ \sigma _{xy}^{\text {**}} \\ q_y^{\text {**}} \\ \end{array} \right) +\left( \begin{array}{l} 0 \\ 0 \\ A_{31} \theta ^{*} {\theta ^{*}}' \\ A_{32} \theta ^{*} {\theta ^{*}}' \\ 0 \\ A_{33} {\theta ^{*}}{}^2 \\ \end{array} \right) } \end{aligned}$$

or

45 $$\begin{aligned} \left( \begin{array}{l} D v_x^{\text {**}} \\ D v_y^{\text {**}} \\ D \theta ^{\text {**}} \\ D \sigma _{yy}^{\text {**}} \\ D \sigma _{xy}^{\text {**}} \\ D q_y^{\text {**}} \\ \end{array} \right)= & {} \left( \begin{array}{llllll} 0 &{} A_{16} &{} 0 &{} 0 &{} A_{17} &{} 0 \\ A_{18} &{} 0 &{} 0 &{} A_{19} &{} 0 &{} 0 \\ 0 &{} 0 &{} 0 &{} 0 &{} 0 &{} A_{20} \\ 0 &{} A_{21} &{} 0 &{} 0 &{} A_{16} &{} A_{22} \\ A_{23} &{} 0 &{} A_{24} &{} A_{18} &{} 0 &{} 0 \\ A_{25} &{} 0 &{} A_{26} &{} A_{27} &{} 0 &{} 0\text { } \\ \end{array} \right) \left( \begin{array}{l} v_x^{\text {**}} \\ v_y^{\text {**}} \\ \theta ^{\text {**}} \\ \sigma _{yy}^{\text {**}} \\ \sigma _{xy}^{\text {**}} \\ q_y^{\text {**}} \\ \end{array} \right) \nonumber \\{} & {} - \left( \begin{array}{l} 0 \\ 0 \\ \sum _{i=1}^3 \sum _{j=1}^3 A_{31} \xi _{ih} v_{3 i h} v_{3 j h} M_{ih} M_{jh} \, e^{-y \left( \xi _{ih}+\xi _{jh}\right) } \\ \sum _{i=1}^3 \sum _{j=1}^3 A_{32} \xi _{ih} v_{3 i h} v_{3 j h} M_{ih} M_{jh} \, e^{-y \left( \xi _{ih}+\xi _{jh}\right) } \\ 0 \\ \sum _{i=1}^3 \sum _{j=1}^3 -A_{33} v_{3 i h} v_{3 j h} M_{ih} M_{jh} \, e^{-y \left( \xi _{ih}+\xi _{jh}\right) } \\ \end{array} \right) \end{aligned}$$

Solving the homogenuous part of Eq. (45), one writes down the characteristic polynomial as:

46 $$\begin{aligned} \zeta ^6 _h- C_1 \zeta ^4 _h +C_2 \zeta ^2 _h-C_3=0, \end{aligned}$$

where $C_1, C_2$ and $C_3$ are constants listed in Supplementary Appendix A.

As for the first order solution, only three roots, $\zeta _{nh}, n=1,2,3$ will contribute to the bounded solution of Eq. (45). Now solve for the non-homogeneous part by the method of undetermined coefficients. The particular solution is taken in the form:

47 $$\begin{aligned} \sum _{i=1}^3 \sum _{j=1}^3 E_{nh,ij} v_{3ih} v_{3jh} \, M_{ih} M_{jh} \, e^{-y \left( \xi _{ih} +\xi _{jh}\right) }, \quad n=1,2, \cdots , 6. \end{aligned}$$

Substitution of this expression into the system yields the coefficients $E_{nh,ij}$:

$$\begin{aligned} { \begin{pmatrix} E_{1h,ij} \\ E_{2h,ij}\\ E_{3h,ij} \\ E_{4h,ij} \\ E_{5h,ij} \\ E_{6h,ij} \end{pmatrix} \text {=}\left( \begin{array}{llllll} \xi _{ih}+\xi _{jh} &{} A_{16} &{} 0 &{} 0 &{} A_{17} &{} 0 \\ A_{18} &{} \xi _{ih}+\xi _{jh} &{} 0 &{} A_{19} &{} 0 &{} 0 \\ 0 &{} 0 &{} \xi _{ih}+\xi _{jh} &{} 0 &{} 0 &{} A_{20} \\ 0 &{} A_{21} &{} 0 &{} \xi _{ih}+\xi _{jh} &{} A_{16} &{} A_{22} \\ A_{23} &{} 0 &{} A_{24} &{} A_{18} &{} \xi _{ih}+\xi _{jh} &{} 0 \\ A_{25} &{} 0 &{} A_{26} &{} A_{27} &{} 0 &{} \xi _{ih}+\xi _{jh} \\ \end{array} \right) ^{-1}\left( \begin{array}{l} 0 \\ 0 \\ A_{31} \xi _{ih}\\ A_{32} \xi _{ih}\\ 0 \\ -A_{33} \\ \end{array} \right) }. \end{aligned}$$

Hence the solution of the system of equations (45) has the form:

48 $$\begin{aligned}{} & {} {v_x^{\text {**}}=\sum _{i=1}^3 V_{1 i h} L_{ih}e^{-\zeta _{ih}y}+\sum _{i=1}^3 \sum _{j=1}^3 E_{1h,ij} v_{3 ih} v_{3 jh} M_{ih} M_{jh} \, e^{-\left( \xi _{ih}+\xi _{jh}\right) y}},\end{aligned}$$

49 $$\begin{aligned}{} & {} {v_y^{\text {**}}=\sum _{i=1}^3 V_{2 i h} L_{ih}e^{-\zeta _{ih}y}+\sum _{i=1}^3 \sum _{j=1}^3 E_{2h,ij} v_{3 ih} v_{3 jh} M_{ih} M_{jh} \, e^{-\left( \xi _{ih}+\xi _{jh}\right) y}},\end{aligned}$$

50 $$\begin{aligned}{} & {} {\theta ^{\text {**}}=\sum _{i=1}^3 V_{3 i h} L_{ih}e^{-\zeta _{ih}y}+\sum _{i=1}^3 \sum _{j=1}^3 E_{3h,ij} v_{3 ih} v_{3 jh} M_{ih} M_{jh} \, e^{-\left( \xi _{ih}+\xi _{jh}\right) y}},\end{aligned}$$

51 $$\begin{aligned}{} & {} {\sigma _{yy}^{\text {**}}=\sum _{i=1}^3 V_{4 i h} L_{ih}e^{-\zeta _{ih}y}+\sum _{i=1}^3 \sum _{j=1}^3 E_{4h,ij} v_{3 ih} v_{3 jh} M_{ih} M_{jh} \, e^{-\left( \xi _{ih}+\xi _{jh}\right) y}},\end{aligned}$$

52 $$\begin{aligned}{} & {} {\sigma _{xy}^{\text {**}}=\sum _{i=1}^3 V_{5 i h} L_{ih}e^{-\zeta _{ih}y}+\sum _{i=1}^3 \sum _{j=1}^3 E_{5h,ij} v_{3 ih} v_{3 jh} M_{ih} M_{jh} \, e^{-\left( \xi _{ih}+\xi _{jh}\right) y}},\end{aligned}$$

53 $$\begin{aligned}{} & {} {q_y^{\text {**}}=\sum _{i=1}^3 V_{6 i h} L_{ih}e^{-\zeta _{ih}y}+\sum _{i=1}^3 \sum _{j=1}^3 E_{6h,ij} v_{3 ih} v_{3 jh} M_{ih} M_{jh} \, e^{-\left( \xi _{ih}+\xi _{jh}\right) y}}. \end{aligned}$$

where $V_{nj h}, \, n=1,2,...,6, \, j=1,2,3$ are constants listed in Supplementary Appendix D.

#### Slab

$$\begin{aligned} {\left( \begin{array}{l} D v_x^{\text {**}} \\ D v_y^{\text {**}} \\ D \theta ^{\text {**}} \\ D \sigma _{yy}^{\text {**}} \\ D \sigma _{xy}^{\text {**}} \\ D q_y^{\text {**}} \\ \end{array} \right) =\left( \begin{array}{llllll} 0 &{} A_{16} &{} 0 &{} 0 &{} A_{17} &{} 0 \\ A_{18} &{} 0 &{} 0 &{} A_{19} &{} 0 &{} 0 \\ 0 &{} 0 &{} 0 &{} 0 &{} 0 &{} A_{20} \\ 0 &{} A_{21} &{} 0 &{} 0 &{} A_{16} &{} A_{22} \\ A_{23} &{} 0 &{} A_{24} &{} A_{18} &{} 0 &{} 0 \\ A_{25} &{} 0 &{} A_{26} &{} A_{27} &{} 0 &{} 0\text { } \\ \end{array} \right) \left( \begin{array}{l} v_x^{\text {**}} \\ v_y^{\text {**}} \\ \theta ^{\text {**}} \\ \sigma _{yy}^{\text {**}} \\ \sigma _{xy}^{\text {**}} \\ q_y^{\text {**}} \\ \end{array} \right) +\left( \begin{array}{l} 0 \\ 0 \\ A_{31} \theta ^{*} {\theta ^{*}}' \\ A_{32} \theta ^{*} {\theta ^{*}}' \\ 0 \\ A_{33} {\theta ^{*}}{}^2 \\ \end{array} \right) } \end{aligned}$$

or

54 $$\begin{aligned} \left( \begin{array}{l} D v_x^{\text {**}} \\ D v_y^{\text {**}} \\ D \theta ^{\text {**}} \\ D \sigma _{yy}^{\text {**}} \\ D \sigma _{xy}^{\text {**}} \\ D q_y^{\text {**}} \\ \end{array} \right)= & {} \left( \begin{array}{llllll} 0 &{} A_{16} &{} 0 &{} 0 &{} A_{17} &{} 0 \\ A_{18} &{} 0 &{} 0 &{} A_{19} &{} 0 &{} 0 \\ 0 &{} 0 &{} 0 &{} 0 &{} 0 &{} A_{20} \\ 0 &{} A_{21} &{} 0 &{} 0 &{} A_{16} &{} A_{22} \\ A_{23} &{} 0 &{} A_{24} &{} A_{18} &{} 0 &{} 0 \\ A_{25} &{} 0 &{} A_{26} &{} A_{27} &{} 0 &{} 0\text { } \\ \end{array} \right) \left( \begin{array}{l} v_x^{\text {**}} \\ v_y^{\text {**}} \\ \theta ^{\text {**}} \\ \sigma _{yy}^{\text {**}} \\ \sigma _{xy}^{\text {**}} \\ q_y^{\text {**}} \\ \end{array} \right) \nonumber \\{} & {} + \left( \begin{array}{l} 0 \\ 0 \\ \sum _{i=1}^3 \sum _{j=1}^3 A_{31} ( - \xi _{i s} v_{3 i s} v_{3 j s} M_{i s} M_{j s} \, e^{-y \left( \xi _{i s}+\xi _{j s}\right) } \\ +(-\xi _{i s}+\xi _{j s}) v_{3 i s} V_{3 j s} M_{i s} m_{j s} \, e^{y \left( -\xi _{i s}+\xi _{j s}\right) } \\ +\xi _{i s} V_{3 i s} V_{3 j s} m_{i s} m_{j s} \, e^{y \left( \xi _{i s}+\xi _{j s}\right) } ) \\ \sum _{i=1}^3 \sum _{j=1}^3 A_{32} (v_{3 i s} v_{3 j s} M_{i s} M_{j s} \, e^{-y \left( \xi _{i s}+\xi _{j s}\right) } \\ +( 2 v_{3 i s} V_{3 j s} M_{i s} m_{j s} \, e^{y \left( -\xi _{i s}+\xi _{j s}\right) } \\ V_{3 i s} V_{3 j s} m_{i s} m_{j s} \, e^{y \left( \xi _{i s}+\xi _{j s}\right) } ) \\ 0 \\ \sum _{i=1}^3 \sum _{j=1}^3 A_{33} ( - \xi _{i s} v_{3 i s} v_{3 j s} M_{i s} M_{j s} \, e^{-y \left( \xi _{i s}+\xi _{j s}\right) } \\ +(-\xi _{i s}+\xi _{j s}) v_{3 i s} V_{3 j s} M_{i s} m_{j s} \, e^{y \left( -\xi _{i s}+\xi _{j s}\right) } \\ +\xi _{i s} V_{3 i s} V_{3 j s} m_{i s} m_{j s} \, e^{y \left( \xi _{i s}+\xi _{j s}\right) } ) \\ \end{array} \right) \end{aligned}$$

Solving the homogenuous part of Eq. (54), one writes down the characteristic polynomial as:

55 $$\begin{aligned} \zeta _s ^6- C_1 \zeta _s^4+C_2 \zeta _s^2-C_3=0. \end{aligned}$$

Now solve for the non-homogeneous part by the method of undetermined coefficients. The particular solution is taken in the form:

$$\begin{aligned} \sum _{i=1}^3 \sum _{j=1}^3( Q_{n,ij} v_{3 is} v_{3 js} \, M_{i s} M_{j s} \, e^{-y \left( \xi _{i s}+\xi _{j s}\right) }\\ +W_{n,ij} v_{3 i s} V_{3 j s} M_{i s} m_{j s} \, e^{y \left( -\xi _{i s}+\xi _{j s}\right) } \\ +O_{n,ij} V_{3 i s} V_{3 j s} m_{i s} m_{j s} \, e^{y \left( \xi _{i s}+\xi _{j s}\right) }), \quad n=1,2, \cdots , 6. \end{aligned}$$

Substitution of this expression into the system yields the coefficients $Q_{n,ij}$,$W_{n,ij}$ and $O_{n,ij}$ are given in Supplementary Appendix G.

Hence the solution of the system of equations (54) has the form:

$$\begin{aligned} v_x^{\text {**}}=\sum _{i=1}^3 \text { }u_{1 is}L_{is} e^{-\zeta _{i s}y}+ \sum _{i=1}^3 \text { }U_{1 is}Y_{is} e^{\zeta _{i s}y}+ \sum _{i=1}^3 \sum _{j=1}^3( Q_{1,ij} v_{3 is} v_{3 js} \, M_{i s} M_{j s} \, e^{-y \left( \xi _{i s}+\xi _{j s}\right) }\\ +W_{1,ij} v_{3 i s} V_{3 j s} M_{i s} m_{j s} \, e^{y \left( -\xi _{i s}+\xi _{j s}\right) } +O_{1,ij} V_{3 i s} V_{3 j s} m_{i s} m_{j s} \, e^{y \left( \xi _{i s}+\xi _{j s}\right) }), \\ v_y^{\text {**}}=\sum _{i=1}^3 \text { }u_{2 is}L_{is} e^{-\zeta _{i s}y}+ \sum _{i=1}^3 \text { }U_{2 is}Y_{is} e^{\zeta _{i s}y}+ \sum _{i=1}^3 \sum _{j=1}^3( Q_{2,ij} v_{3 is} v_{3 js} \, M_{i s} M_{j s} \, e^{-y \left( \xi _{i s}+\xi _{j s}\right) }\\ +W_{2,ij} v_{3 i s} V_{3 j s} M_{i s} m_{j s} \, e^{y \left( -\xi _{i s}+\xi _{j s}\right) } +O_{2,ij} V_{3 i s} V_{3 j s} m_{i s} m_{j s} \, e^{y \left( \xi _{i s}+\xi _{j s}\right) }), \\ \theta ^{\text {**}}=\sum _{i=1}^3 \text { }u_{3 is}L_{is} e^{-\zeta _{i s}y}+ \sum _{i=1}^3 \text { }U_{3 is}Y_{is} e^{\zeta _{i s}y}+ \sum _{i=1}^3 \sum _{j=1}^3( Q_{3,ij} v_{3 is} v_{3 js} \, M_{i s} M_{j s} \, e^{-y \left( \xi _{i s}+\xi _{j s}\right) }\\ +W_{3,ij} v_{3 i s} V_{3 j s} M_{i s} m_{j s} \, e^{y \left( -\xi _{i s}+\xi _{j s}\right) } +O_{3,ij} V_{3 i s} V_{3 j s} m_{i s} m_{j s} \, e^{y \left( \xi _{i s}+\xi _{j s}\right) }), \\ \sigma _{yy}^{\text {**}}=\sum _{i=1}^3 \text { }u_{4 is}L_{is} e^{-\zeta _{i s}y}+ \sum _{i=1}^3 \text { }U_{4 is}Y_{is} e^{\zeta _{i s}y}+ \sum _{i=1}^3 \sum _{j=1}^3( Q_{4,ij} v_{3 is} v_{3 js} \, M_{i s} M_{j s} \, e^{-y \left( \xi _{i s}+\xi _{j s}\right) }\\ +W_{4,ij} v_{3 i s} V_{3 j s} M_{i s} m_{j s} \, e^{y \left( -\xi _{i s}+\xi _{j s}\right) } +O_{4,ij} V_{3 i s} V_{3 j s} m_{i s} m_{j s} \, e^{y \left( \xi _{i s}+\xi _{j s}\right) }), \\ \sigma _{xy}^{\text {**}}=\sum _{i=1}^3 \text { }u_{5 is}L_{is} e^{-\zeta _{i s}y}+ \sum _{i=1}^3 \text { }U_{5 is}Y_{is} e^{\zeta _{i s}y}+ \sum _{i=1}^3 \sum _{j=1}^3( Q_{5,ij} v_{3 is} v_{3 js} \, M_{i s} M_{j s} \, e^{-y \left( \xi _{i s}+\xi _{j s}\right) }\\ +W_{5,ij} v_{3 i s} V_{3 j s} M_{i s} m_{j s} \, e^{y \left( -\xi _{i s}+\xi _{j s}\right) } +O_{5,ij} V_{3 i s} V_{3 j s} m_{i s} m_{j s} \, e^{y \left( \xi _{i s}+\xi _{j s}\right) }), \\ q _{y}^{\text {**}}=\sum _{i=1}^3 \text { }u_{6 is}L_{is} e^{-\zeta _{i s}y}+ \sum _{i=1}^3 \text { }U_{6 is}Y_{is} e^{\zeta _{i s}y}+ \sum _{i=1}^3 \sum _{j=1}^3( Q_{6,ij} v_{3 is} v_{3 js} \, M_{i s} M_{j s} \, e^{-y \left( \xi _{i s}+\xi _{j s}\right) }\\ +W_{6,ij} v_{3 i s} V_{3 j s} M_{i s} m_{j s} \, e^{y \left( -\xi _{i s}+\xi _{j s}\right) } +O_{6,ij} V_{3 i s} V_{3 j s} m_{i s} m_{j s} \, e^{y \left( \xi _{i s}+\xi _{j s}\right) }), \end{aligned}$$

where $U_{njs}$ and $u_{njs}$, $n=1,2,...,6, \, j=1,2,3$ are constants listed in Supplementary Appendix E.

#### Boundary conditions for the non-homogeneous system

At the interface ($y=d$):

56a $$\begin{aligned} v_{xs}&= v_{xh}, \end{aligned}$$

56b $$\begin{aligned} v_{ys}&= v_{yh}, \end{aligned}$$

56c $$\begin{aligned} \sigma _{xys}&= \sigma _{xyh}, \end{aligned}$$

56d $$\begin{aligned} \sigma _{yys}&= \sigma _{yyh}, \end{aligned}$$

56e $$\begin{aligned} K_{s} \theta _{s}&= K_{0} \theta _{h} \end{aligned}$$

56f $$\begin{aligned} K_{s} \theta _{,ys}&= K_{0} \theta _{,yh} \end{aligned}$$

At the outer boundary ($y=0$): Vanishing boundary conditions prevail, since the solution at this order of approximation is generated by the non-homogeneous term from the previous order of approximation. :

57a $$\begin{aligned} \sigma _{xys}&= 0, \end{aligned}$$

57b $$\begin{aligned} \sigma _{yys}&= 0, \end{aligned}$$

57c $$\begin{aligned} \theta _{s}&= 0. \end{aligned}$$

Applying the boundary conditions to determine the $L_{ih}$ , $L_{is}$ and $Y_{is}$, one gets the system of equations given in Supplementary Appendix I. This system is cast in the following matricial form

58 $$\begin{aligned} \begin{pmatrix} L_{s 1} \\ L_{s 2} \\ L_{s 3} \\ Y_{s 1} \\ Y_{s 2} \\ Y_{s 3} \\ L_{ 1} \\ L_{ 2} \\ L_{3} \\ \end{pmatrix}=\begin{pmatrix} \Lambda _{10} &{} \Lambda _{11} &{} \Lambda _{12} \\ \Lambda _{13}&{} \Lambda _{14} &{} \Lambda _{15}\\ \Lambda _{16}&{}\Lambda _{17} &{} \Lambda _{18} \\ \end{pmatrix}^{-1}\begin{pmatrix} g_{1}\\ g_2\\ g_3\\ g_4\\ g_5 \\ g_6 \\ g_7 \\ g_8 \\ g_9 \\ \end{pmatrix} \end{aligned}$$

where $\Lambda _{10}-\Lambda _{18}$ and $g_1-g_9$ are matrices given in Supplementary Appendix F.

## Numerical results and discussion

This section is devoted to the analysis of a concrete numerical example. As noted earlier, we have preferred, for the sake of brevity, not to deal with the dispersion relation which is directly obtained as the condition of existence of solution for the obtained system of algebraic equations in the first order of approximation. Rather, we have chosen specific, otherwise arbitrary, values for the frequency, wave number and attenuation coefficient to proceed with the numerical calculations.

The following values were taken for the prescribed temperature and the pressure at the external boundary:

$$\begin{aligned} f^*_1=0, \qquad f^*_2=-0.5., \qquad f^*_3=1.0, \qquad \eta =1.0,\qquad d=1. \end{aligned}$$

The materials chosen for the purpose of numerical calculations are taken as Cadmium Selenide (CdSe) and Lead Zirconate Titanate (PZT-5A), both having hexagonal symmetry (6 mm class). Although this material has piezoelectric properties, it was chosen for its thermoelastic characteristics only, while all thermoelectric and electromechanical couplings were disregarded. The following values of the different material parameters are chosen as in (Sharma and Kumar^21^; Walia et al.^22^). The coordinate *z*-axis was oriented perpendicular to the plane of symmetry. The corresponding values of the material parameters are shown in Tables 1 and 2 (c.f.^18^):

**Table 1:** Values of the geometrical and the material parameters for the half-space.

$\Theta _0=298 \, \textrm{K}$	$K_0=9 \, \textrm{W m}^{-1}\, \textrm{K}^{-1}$	
$\rho =5504 \, \textrm{kg} \, \textrm{m}^{-3}$	$C_e =260 \, \textrm{J} \, \textrm{kg}^{-1} \, \textrm{K}^{-1}$	$\gamma =0.5 \times 10^5 \, \textrm{m}^3 \, \textrm{kg}^{-1}$
$\lambda =2.6 \times 10^{10} \, \textrm{kg m}^{-1}\, s^{-2}$	$\mu =1.6 \times 10^{10} \, \textrm{kg m}^{-1}\, s^{-2}$	
$\tau _0=1.000 \times 10^{-11} \textrm{s}$	$\tau _{\theta }=0.500 \times 10^{-13 } \textrm{s}$	$\tau _q=0.600 \times 10^{-11} \textrm{s}$
$k=0.2 \, \text {(wave \, number)}$	$\omega _r=0.5$	$\omega _i=0.5$

**Table 2:** Values of the geometrical and the material parameters for the slab.

$\Theta _0=298 \, \textrm{K}$	$K_s=1.5 \, \textrm{W} \textrm{m}^{-1} \textrm{K}^{-1}$	
$\rho _s =7750 \, \mathrm{kg\, m}^{-3}$	$C_e =260 \, \mathrm{J \, kg}^{-1} \textrm{K}^{-1}$	$\gamma _s =0.2 \times 10^5 \, \textrm{m}^3 \, \textrm{kg}^{-1}$
$\lambda _s=0.8 \times 10^{10} \, \textrm{kg m}^{-1} \textrm{s}^{-2}$	$\mu _s =0.6 \times 10^{10} \, \mathrm{kg \, m}^{-1} \textrm{s}^{-2}$	
$\tau _0=1.000 \times 10^{-11} \textrm{s}$	$\tau _{\theta }=0.500 \times 10^{-13 } \textrm{s}$	$\tau _q=0.600 \times 10^{-11} \textrm{s}$
$k=0.2 \, \text {(wave\, number)}$	$\omega _r=0.5$	$\omega _i=0.5$

The numerical data have been presented in three sets of plots. The first one concerns the solution at the first order (linear) of approximation, the second one is for the second order solution for three values of the nonlinearity parameter $\eta$, all evaluated at a particular location at a particular time moment. As to the third set of plots, it is intended for the comparison of solutions in the half-space with/without the bonded slab. All calculations were carried out using the Software package Mathematica 13.2.

**Figure 2:**
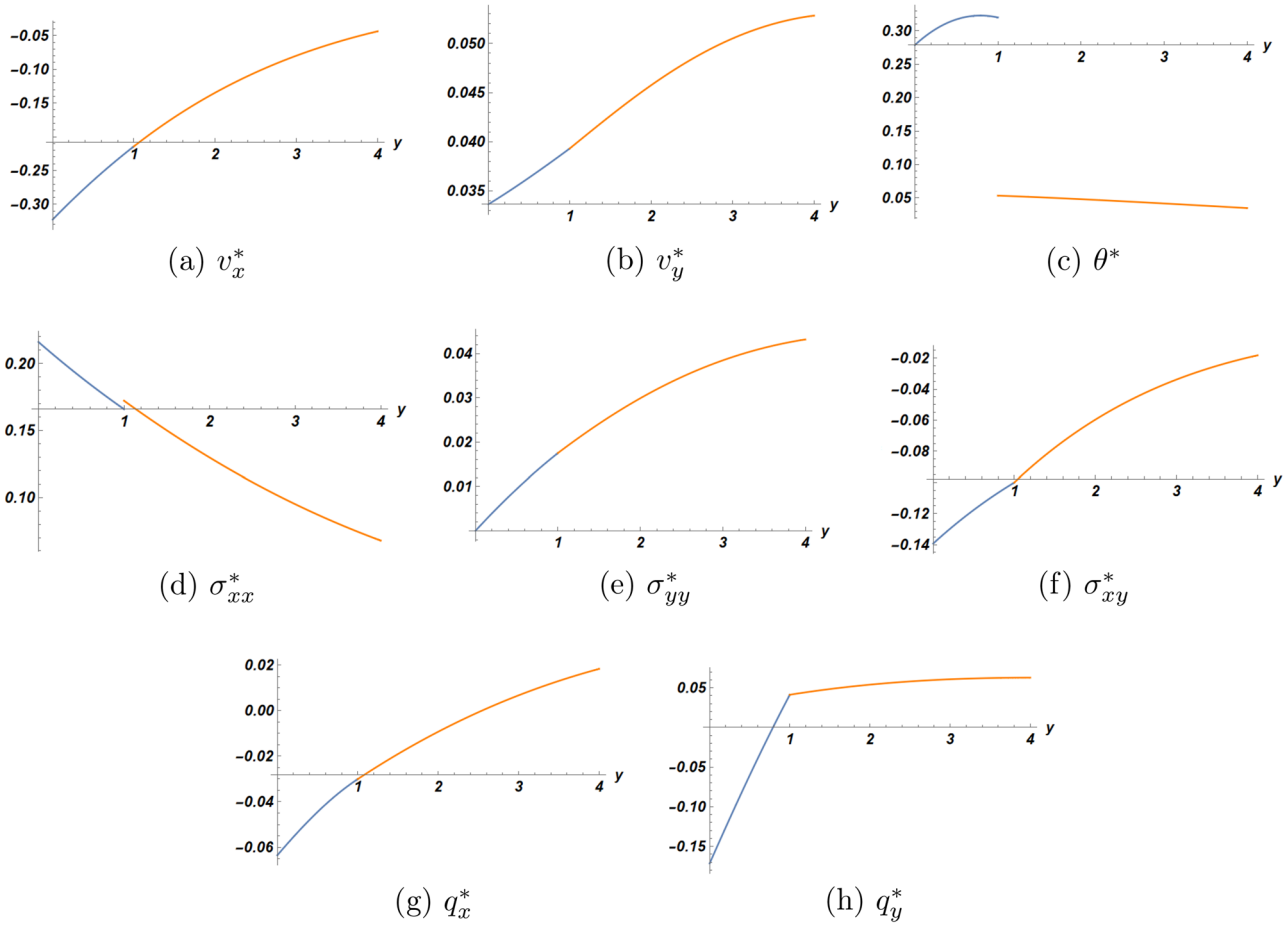
First order solution as function of *y* at $x=2.0$ and $t=2.1$ (blue dot) half-space (orange dot) slab.

Plots in Fig. 2 for the solution at the first order of approximation as function of *y* at the location $x=2.0$ and at time $t=2.1$ describe the behavior of the solution in the interval $0 \le y \le 4$ including the interface. It shows the continuity of both velocity components at the interface, as should be. This is satisfied in all coming figures. However, there exists a jump for both the temperature and the stress component $\sigma _{xx}$ at the interface, while all the other functions are continuous there. The heat flux component $q_y$ seems to have a jump in the first-order derivative. As noted above, the jump in temperature is inherent to the used model of DPL and can be used to evaluate one material coefficient of the slab from experiment.

**Figure 3:**
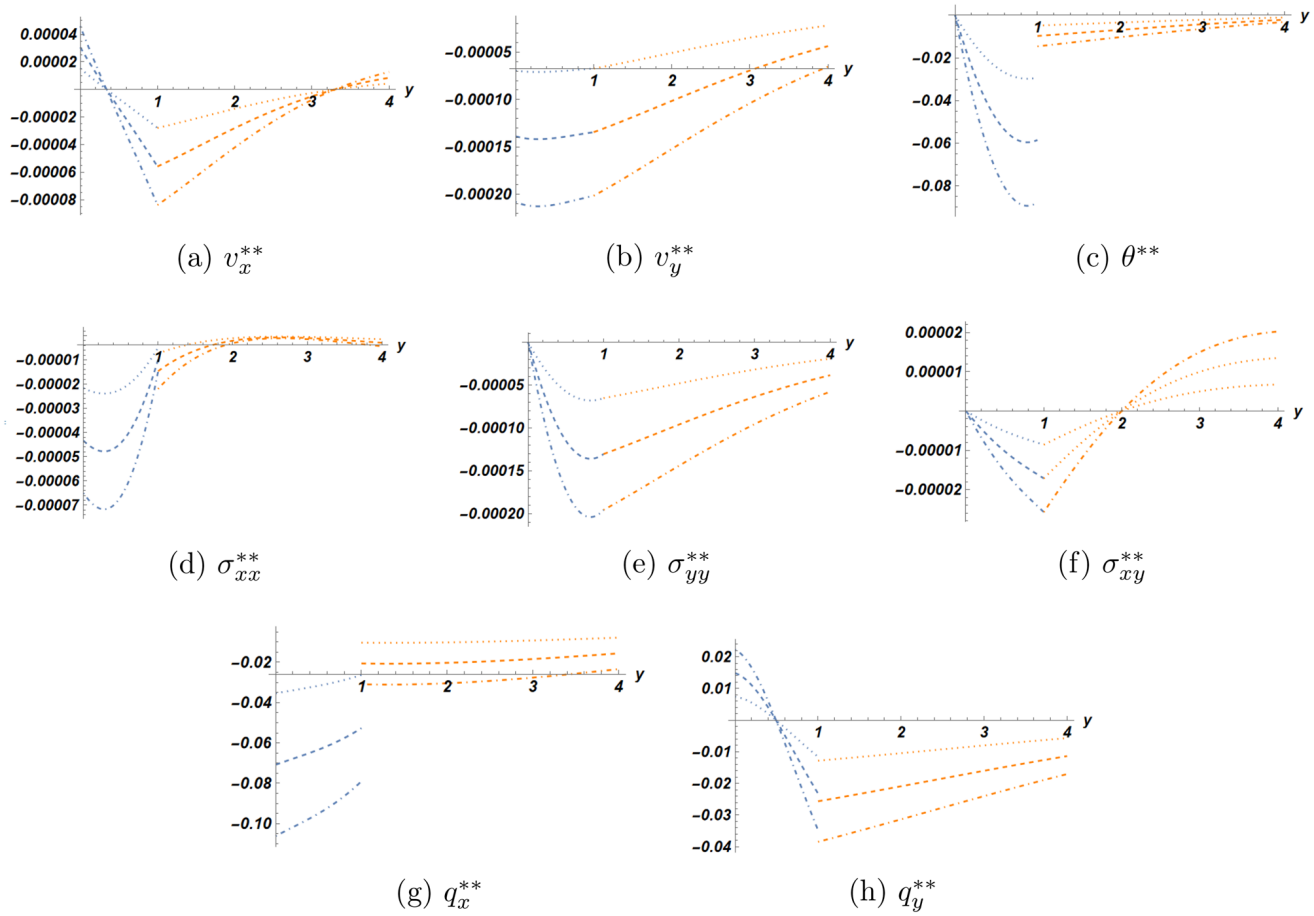
Second order solution as function of *y* at $x=2.0$, $t=2.1$ for three values of $\eta$ (blue dot) half-space (orange dot) slab (blue dot line) $\eta =1$ (orange dashed line) $\eta =2$ (orange dashed line with dots) $\eta =3$.

In Fig. 3 for the second order solution, the plots show a different behaviour of the heat flux than in the first order solution. In fact, both components $q_x, q_y$ have now got jumps at the interface, that can be easily identified for all three values of the parameter $\eta$. Moreover, the absolute value of the jump increases with the increase of the nonlinearity parameter $\eta$. The existence of discontinuous heat flux at the interface constitutes a major difference between extended thermodynamics and classical thermodynamics for which Fourier law for heat conduction is valid.

**Figure 4:**
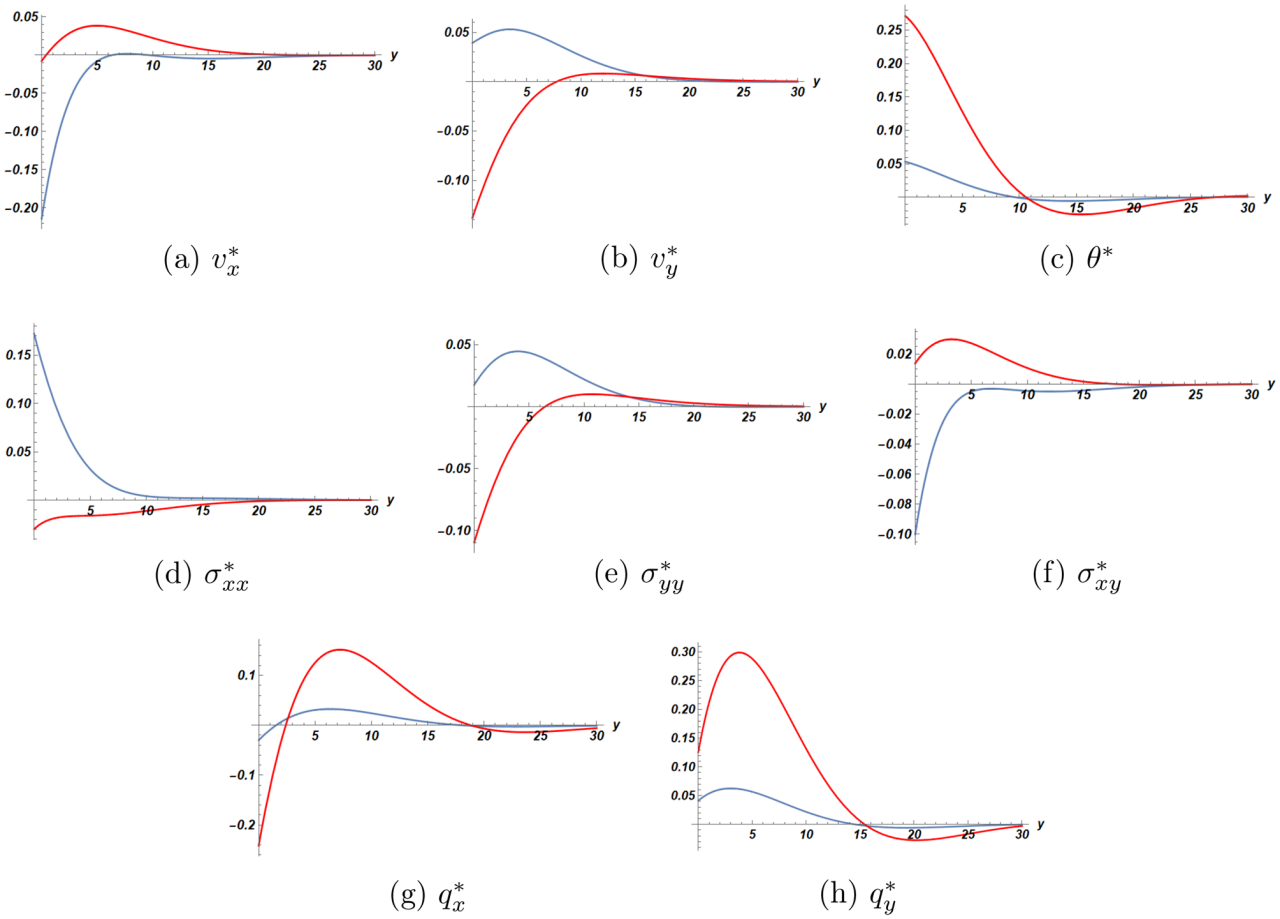
Comparison of first order solutions as functions of *y* at $x=2$, $t=2.1$ (red dot) half-space (blue dot) half-space with slab.

**Figure 5:**
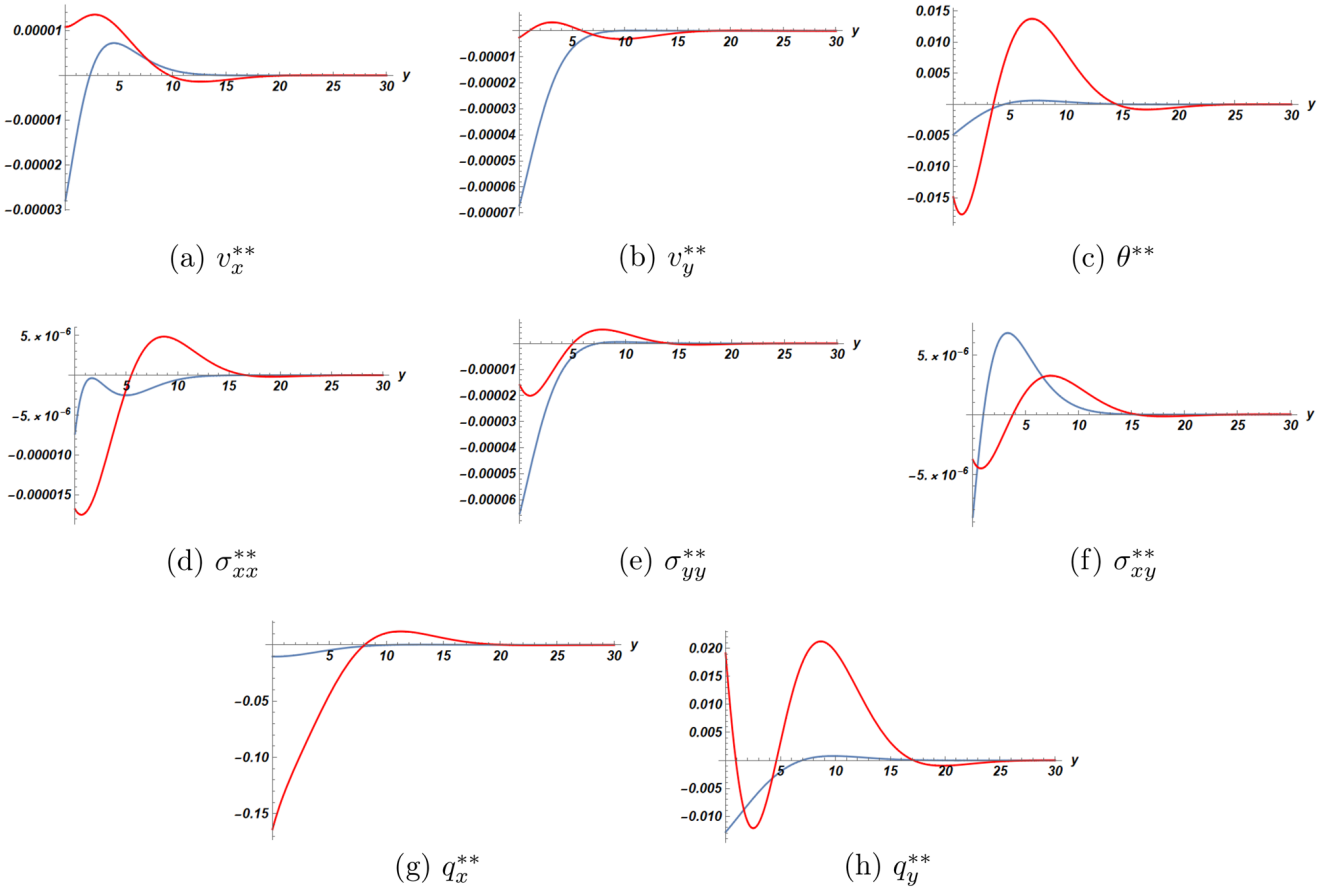
Comparison of second order solutions as functions of *y* at $x=2$, $t=2.1$ (red dot) half-space (blue dot) half-space with slab.

Figures 4 and 5 are devoted to the comparison of solutions at the first and the second orders of approximation respectively between the cases of a half-space with/without slab. The results for the half-space without slab were recently published by the same authors^20^. According to these figures, we concluded that the presence of the bonded slab yields faster attenuation of the solution with depth.

## Conclusions

An earlier work by the authors^20^ has been extended to the case of a layered thermoelastic medium consisting of a thick layer rigidly bonded to a half-space. On one hand, this allows to assess the effect of the slab on wave propagation in the half-space. On the other hand, such a layered medium can be used to measure some material coefficients of the slab when the characteristics of the half-space (matrix) are known. Here, both media have temperature dependence of the thermal conductivity, thus leading to a nonlinear problem. All other nonlinear couplings were disregarded in the governing system of equations. Nonlinear Rayleigh wave propagation is used to test the response of the layer. At the interface, in addition to the conditions of bonding, the other boundary conditions are obtained from the field equations by well-known rules of Continuum Mechanics. Poincaré expansion of the solution in a small parameter in the first two orders of approximation yields expressions which are adequate for the description of wave propagation in both media and allows to evaluate the coefficient of the nonlinear coupling in the slab through heat wave propagation measurement. It is emphasized that this is an approximate particular solution only. Other solutions may potentially be obtained through different methods, depending on the nature of the considered medium and the phenomenon to be explained. For the considered numerical values of the different material coefficients, the proposed plots show that the temperature and one stress component along the direction of wave propagation suffer jumps at the interface, while the other stress components are continuous there. The heat flux components are continuous at the interface at the first approximation, but appear to have jumps there at the second approximation. These jumps at the interface may used to determine some material coefficients in conjunction with measurements. Comparison was carried out with the case of a half-space recently published by the authors. It is shown that the presence of a thick slab bonded to the half-space favours faster attenuation with depth of all the functions in the half-space.

## Supplementary Information

Supplementary Information.

## Data Availability

All data generated or analyzed during this study are included in this article
