# Supplementary material for: Nonlinear Rayleigh wave propagation in a layered half-space in dual-phase-lag
Source: Sci Rep. 2023 Feb 7;13:2187. doi: 10.1038/s41598-023-29411-4 (PMC9905529; doi:10.1038/s41598-023-29411-4)
Supplement: Supplementary file 1 — Supplementary Information. [file 41598_2023_29411_MOESM1_ESM.pdf]

Appendix A

Coefficients 1

$A_1 = -ik, A_2 = -\omega_r - i\omega_i, A_3 = \frac{-ik\alpha}{\beta}, A_4 = \frac{(\alpha-\beta)(\alpha+\beta)(\omega_r+i\omega_i)}{\beta},$

$A_5 = \frac{1-(\omega_r+i\omega_i)\tau_q}{K_0(-1+(\omega_r+i\omega_i)\tau_\theta)}, A_6 = -\frac{(\omega_r+i\omega_i)}{\beta_1}, A_7 = -\frac{\beta_4(-1+(\omega_r+i\omega_i)\tau_q)}{K_0(-1+(\omega_r+i\omega_i)\tau_\theta)},$

$A_8 = -\frac{k^2}{\beta(\omega_r+i\omega_i)} - \frac{(\omega_r+i\omega_i)}{\beta_1}, A_9 = ik\beta_4, A_{11} = \left((\omega_r + i\omega_i) - \frac{k^2 K_0(-1+(\omega_r+i\omega_i)\tau_\theta)}{-1+(\omega_r+i\omega_i)\tau_q}\right),$

$A_{10} = \frac{(\alpha-\beta)\beta_2(ik)}{\beta}, A_{12} = \frac{(-\alpha+\beta)(\alpha+\beta)(\omega_r+i\omega_i)\beta_2}{\beta}, A_{13} = \frac{k}{\beta(i\omega_r-\omega_i)}, A_{14} = \frac{\alpha}{\beta},$

$A_{15} = \frac{kK_0(i+(-i\omega_r+\omega_i)\tau_\theta)}{-1+(\omega_r+i\omega_i)\tau_q}, A_{16} = -i2k, A_{17} = -2\left(\omega_r + i\omega_i\right), A_{18} = -\frac{i2\ k\alpha}{\beta},$

$A_{19} = \left(-2\beta\left(\omega_r + i\omega_i\right)\right) + \frac{\alpha(2\alpha(\omega_r+i\omega_i))}{\beta}, A_{20} = \frac{(1-2(\omega_r+i\omega_i)\tau_q)}{K_0(-1+2(\omega_r+i\omega_i)\tau_\theta)},$

$A_{21} = -\frac{2(\omega_r+i\omega_i)}{\beta_1}, A_{22} = \frac{\beta_4(1-2(\omega_r+i\omega_i)\tau_q)}{K_0(-1+2(\omega_r+i\omega_i)\tau_\theta)}, A_{23} = -\frac{2k^2}{\beta(\omega_r+i\omega_i)} - \frac{2(\omega_r+i\omega_i)}{\beta_1},$

$A_{24} = 2ik\beta_4, A_{25} = \frac{2ik(\alpha-\beta)\beta_2}{\beta}, A_{26} = 2\left(\omega_r + i\omega_i\right) - \frac{4k^2 K_0(-1+2(\omega_r+i\omega_i)\tau_\theta)}{-1+2(\omega_r+i\omega_i)\tau_q},$

$A_{27} = -\frac{2\beta_2(\alpha-\beta)(\alpha+\beta)(\omega_r+i\omega_i)}{\beta}, A_{28} = -\frac{ik}{\beta(\omega_r+i\omega_i)}, A_{29} = \frac{\alpha}{\beta},$

$A_{30} = \frac{2kK_0(1+\tau_\theta(-2i\omega_i-2\omega_r))}{i+2\tau_q(\omega_i-i\omega_r)}, A_{31} = \eta\frac{1}{2}\left(-1 + \frac{1}{-1+2\tau_\theta(i\omega_i+\omega_r)}\right), A_{32} = \eta\frac{1}{2}\beta_4\left(-1 + \frac{1}{-1+2\tau_\theta(i\omega_i+\omega_r)}\right),$

$A_{33} = -\eta\frac{2k^2 K_0(-1+\tau_\theta(i\omega_i+\omega_r))}{-1+2\tau_q(i\omega_i+\omega_r)}, A_{34} = \eta\frac{kK_0(1+\tau_\theta(-i\omega_i-\omega_r))}{i+2\tau_{\mathrm{q}}(\omega_i-i\omega_r)},$

$A = \left(2A_1A_3 + A_{11}A_5 + A_4A_6 + A_{12}A_7 + A_2A_8\right),$

$B = A_1^2\left(A_3^2 - A_4A_8\right) + A_4A_5A_6A_{11} + A_1\left(2A_3A_5A_{11} - A_4A_7A_{10} + \left(A_3A_7 - A_5A_9\right)A_{12}\right)$

$-A_2\left(A_3^2A_6 + A_3A_7A_{10} + A_5A_9A_{10} - A_8\left(A_4A_6 + A_5A_{11} + A_7A_{12}\right)\right),$

$C = A_5\left({A_1}^2 - A_2A_6\right)\left(A_{11}\left({A_3}^2 - A_4A_8\right) - A_{12}A_3A_9 + A_{10}A_4A_9\right),$

$C_1 = \left(2A_{16}A_{18} + A_{26}A_{20} + A_{19}A_{21} + A_{27}A_{22} + A_{17}A_{24}\right),$

$C_2 = A_{26}A_{19}A_{20}A_{21} + {A_{16}}^2\left({A_{18}}^2 - A_{19}A_{24}\right)$

$-A_{17}\left({A_{18}}^2A_{21} + A_{25}A_{18}A_{22} - \left(A_{26}A_{20} + A_{19}A_{21} + A_{27}A_{22}\right)A_{24} + A_{25}A_{20}\ A_{24}\right)$

$+A_{16}\left(2A_{26}A_{18}A_{20} + A_{27}A_{18}A_{22} - A_{25}A_{19}A_{22} - A_{27}A_{20}\ A_{24}\right),$

$C_3 = A_{20}\left({A_{16}}^2 - A_{17}A_{21}\right)\left(A_{26}\left({A_{18}}^2 - A_{19}A_{23}\right) - A_{27}A_{18}A_{24} + A_{25}A_{19}A_{24}\right).$

Appendix B

Coefficients 2

$$
\begin{aligned}
v_{1nh} &= \frac{A_1^2A_4A_5A_9 - A_2A_4A_5A_6A_9 + A_2A_3A_7\xi_{nh}^2 + A_1A_4A_7\xi_{nh}^2 + A_2A_5A_9\xi_{nh}^2}{A_5\Delta_{nh}},\\
v_{2nh} &= \frac{\xi_{nh}(-A_2A_3^2A_7 + A_2A_4A_7A_8 - A_2A_3A_5A_9 - A_1A_4A_5A_9 - A_4A_7\xi_{nh}^2)}{A_5\Delta_{nh}},\\
v_{3nh} &= 1.0\\
v_{4nh} &= \frac{-A_1^2A_3A_5A_9 + A_2A_3A_5A_6A_9 - A_1A_3A_7\xi_{nh}^2 - A_2A_7A_8\xi_{nh}^2 + A_1A_5A_9\xi_{nh}^2 + A_7\xi_{nh}^4}{A_5\Delta_{nh}},\\
v_{5nh} &= \frac{\xi_{nh}(-A_1A_3^2A_7 + A_1A_4A_7A_8 - A_1A_3A_5A_9 - A_4A_5A_6A_9 + A_3A_7\xi_{nh}^2 + A_5A_9\xi_{nh}^2)}{A_5\Delta_{nh}},\\
v_{6nh} &= \frac{-\xi_{nh}}{A_5},\\
\Delta_{nh} &= A_1^2A_3^2 - A_2A_3^2A_6 - A_1^2A_4A_8 + A_2A_4A_6A_8 - 2A_1A_3\xi_{nh}^2 - A_4A_6\xi_{nh}^2 - A_2A_8\xi_{nh}^2 + \xi_{nh}^4.
\end{aligned}
$$

Appendix C

Coefficients 3

$$
\begin{aligned}
v_{1ns} &= \frac{A_1^2A_4A_5A_9 - A_2A_4A_5A_6A_9 + A_2A_3A_7\xi_{ns}^2 + A_1A_4A_7\xi_{ns}^2 + A_2A_5A_9\xi_{ns}^2}{A_5\Delta_{ns}},\\
v_{2ns} &= \frac{\xi_{ns}(-A_2A_3^2A_7 + A_2A_4A_7A_8 - A_2A_3A_5A_9 - A_1A_4A_5A_9 - A_4A_7\xi_{ns}^2)}{A_5\Delta_{ns}},\\
v_{3ns} &= 1.0\\
v_{4ns} &= \frac{-A_1^2A_3A_5A_9 + A_2A_3A_5A_6A_9 - A_1A_3A_7\xi_{ns}^2 - A_2A_7A_8\xi_{ns}^2 + A_1A_5A_9\xi_{ns}^2 + A_7\xi_{ns}^4}{A_5\Delta_{ns}},\\
v_{5ns} &= \frac{\xi_{ns}(-A_1A_3^2A_7 + A_1A_4A_7A_8 - A_1A_3A_5A_9 - A_4A_5A_6A_9 + A_3A_7\xi_{ns}^2 + A_5A_9\xi_{ns}^2)}{A_5\Delta_{ns}},\\
v_{6ns} &= \frac{-\xi_{ns}}{A_5},\\
V_{1ns} &= \frac{A_1^2A_4A_5A_9 - A_2A_4A_5A_6A_9 + A_2A_3A_7\xi_{ns}^2 + A_1A_4A_7\xi_{ns}^2 + A_2A_5A_9\xi_{ns}^2}{A_5\Delta_{ns}},\\
V_{2ns} &= \frac{\xi_{ns}(A_2A_3^2A_7 - A_2A_4A_7A_8 + A_2A_3A_5A_9 + A_1A_4A_5A_9 - A_4A_7\xi_{ns}^2)}{A_5\Delta_{ns}},\\
V_{3ns} &= 1.0\\
V_{4ns} &= \frac{A_1^2A_3A_5A_9 - A_2A_3A_5A_6A_9 + A_1A_3A_7\xi_{ns}^2 - A_2A_7A_8\xi_{ns}^2 + A_1A_5A_9\xi_{ns}^2 + A_7\xi_{ns}^4}{A_5\Delta_{ns}},\\
V_{5ns} &= \frac{-\xi_{ns}(-A_1A_3^2A_7 + A_1A_4A_7A_8 - A_1A_3A_5A_9 - A_4A_5A_6A_9 + A_3A_7\xi_{ns}^2 + A_5A_9\xi_{ns}^2)}{A_5\Delta_{ns}},\\
V_{6ns} &= \frac{\xi_{ns}}{A_5},\\
\Delta_{ns} &= A_1^2A_3^2 - A_2A_3^2A_6 - A_1^2A_4A_8 + A_2A_4A_6A_8 - 2A_1A_3\xi_{ns}^2 - A_4A_6\xi_{ns}^2 - A_2A_8\xi_{ns}^2 + \xi_{ns}^4.
\end{aligned}
$$

Appendix D

Coefficients 4

$$
\begin{aligned}
V_{1nh} &= \frac{A_{16}^2 A_{19} A_{20} A_{24} - A_{17} A_{19} A_{20} A_{21} A_{24} + A_{17} A_{18} A_{22} \zeta_{nh}^2 + A_{16} A_{19} A_{22} \zeta_{nh}^2 + A_{17} A_{20} A_{24} \zeta_{nh}^2}{A_{20} \Pi_{nh}}, \\
V_{2nh} &= \frac{\zeta_{nh}(-A_{17} A_{18}^2 A_{22} + A_{17} A_{19} A_{22} A_{23} - A_{17} A_{18} A_{20} A_{24} - A_{16} A_{19} A_{20} A_{24} - A_{19} A_{22} \zeta_{nh}^2)}{A_{20} \Pi_{nh}}, \\
V_{3nh} &= 1.0 \\
V_{4nh} &= \frac{-A_{16}^2 A_{18} A_{20} A_{24} + A_{17} A_{18} A_{20} A_{21} A_{24} - A_{16} A_{18} A_{22} \zeta_{nh}^2 - A_{17} A_{22} A_{23} \zeta_{nh}^2 + A_{16} A_{20} A_{24} \zeta_{nh}^2 + A_{22} \zeta_{nh}^4}{A_{20} \Pi_{nh}}, \\
V_{5nh} &= \frac{\zeta_{nh}(-A_{16} A_{18}^2 A_{22} + A_{16} A_{19} A_{22} A_{23} - A_{16} A_{18} A_{20} A_{24} - A_{19} A_{20} A_{21} A_{24} + A_{18} A_{22} \zeta_{nh}^2 + A_{20} A_{24} \zeta_{nh}^2)}{A_{20} \Pi_{nh}}, \\
V_{6nh} &= \frac{-\zeta_{nh}}{A_{20}}, \\
\Pi_{nh} &= A_{16}^2 A_{18}^2 - A_{17} A_{18}^2 A_{21} - A_{16}^2 A_{19} A_{23} + A_{17} A_{19} A_{21} A_{23} - 2 A_{16} A_{18} \zeta_{nh}^2 - A_{19} A_{21} \zeta_{nh}^2 - A_{17} A_{23} \zeta_{nh}^2 + \zeta_{nh}^4.
\end{aligned}
$$

Appendix E

Coefficients 5

$$
\begin{aligned}
u_{1ns} &= \frac{A_{16}^2 A_{19} A_{20} A_{24} - A_{17} A_{19} A_{20} A_{21} A_{24} + A_{17} A_{18} A_{22} \zeta_{ns}^2 + A_{16} A_{19} A_{22} \zeta_{ns}^2 + A_{17} A_{20} A_{24} \zeta_{ns}^2}{A_{20} \Pi_{ns}}, \\
u_{2ns} &= \frac{\zeta_{ns}(-A_{17} A_{18}^2 A_{22} + A_{17} A_{19} A_{22} A_{23} - A_{17} A_{18} A_{20} A_{24} - A_{16} A_{19} A_{20} A_{24} - A_{19} A_{22} \zeta_{ns}^2)}{A_{20} \Pi_{ns}}, \\
u_{3ns} &= 1.0 \\
u_{4ns} &= \frac{-A_{16}^2 A_{18} A_{20} A_{24} + A_{17} A_{18} A_{20} A_{21} A_{24} - A_{16} A_{18} A_{22} \zeta_{ns}^2 - A_{17} A_{22} A_{23} \zeta_{ns}^2 + A_{16} A_{20} A_{24} \zeta_{ns}^2 + A_{22} \zeta_{ns}^4}{A_{20} \Pi_{ns}}, \\
u_{5ns} &= \frac{\zeta_{ns}(-A_{16} A_{18}^2 A_{22} + A_{16} A_{19} A_{22} A_{23} - A_{16} A_{18} A_{20} A_{24} - A_{19} A_{20} A_{21} A_{24} + A_{18} A_{22} \zeta_{ns}^2 + A_{20} A_{24} \zeta_{ns}^2)}{A_{20} \Pi_{ns}}, \\
u_{6ns} &= \frac{-\zeta_{ns}}{A_{20}}, \\
U_{1ns} &= \frac{-A_{16}^2 A_{19} A_{20} A_{24} + A_{17} A_{19} A_{20} A_{21} A_{24} + A_{17} A_{18} A_{22} \zeta_{ns}^2 + A_{16} A_{19} A_{22} \zeta_{ns}^2 + A_{17} A_{20} A_{24} \zeta_{ns}^2}{A_{20} \Pi_{ns}}, \\
U_{2ns} &= \frac{-\zeta_{ns}(-A_{17} A_{18}^2 A_{22} + A_{17} A_{19} A_{22} A_{23} - A_{17} A_{18} A_{20} A_{24} - A_{16} A_{19} A_{20} A_{24} - A_{19} A_{22} \zeta_{ns}^2)}{A_{20} \Pi_{ns}}, \\
U_{3ns} &= 1.0 \\
U_{4ns} &= \frac{A_{16}^2 A_{18} A_{20} A_{24} - A_{17} A_{18} A_{20} A_{21} A_{24} - A_{16} A_{18} A_{22} \zeta_{ns}^2 - A_{17} A_{22} A_{23} \zeta_{ns}^2 + A_{16} A_{20} A_{24} \zeta_{ns}^2 + A_{22} \zeta_{ns}^4}{A_{20} \Pi_{ns}}, \\
U_{5ns} &= \frac{-\zeta_{ns}(-A_{16} A_{18}^2 A_{22} + A_{16} A_{19} A_{22} A_{23} - A_{16} A_{18} A_{20} A_{24} - A_{19} A_{20} A_{21} A_{24} + A_{18} A_{22} \zeta_{ns}^2 + A_{20} A_{24} \zeta_{ns}^2)}{A_{20} \Pi_{ns}}, \\
u_{6ns} &= \frac{\zeta_{ns}}{A_{20}}, \\
\Pi_{ns} &= A_{16}^2 A_{18}^2 - A_{17} A_{18}^2 A_{21} - A_{16}^2 A_{19} A_{23} + A_{17} A_{19} A_{21} A_{23} - 2 A_{16} A_{18} \zeta_{ns}^2 - A_{19} A_{21} \zeta_{ns}^2 - A_{17} A_{23} \zeta_{ns}^2 + \zeta_{ns}^4.
\end{aligned}
$$

Appendix F

Coefficients 6

$$\Lambda_1 = \begin{pmatrix} v_{11s}\ e^{-\xi_{1s}d}\ v_{12s}\ e^{-\xi_{2s}d}\ v_{13s}\ e^{-\xi_{3s}d} \\ v_{21s}\ e^{-\xi_{1s}d}\ v_{22s}\ e^{-\xi_{2s}d}\ v_{23s}\ e^{-\xi_{3s}d} \\ v_{51s}\ e^{-\xi_{1s}d}\ v_{52s}\ e^{-\xi_{2s}d}\ v_{53s}\ e^{-\xi_{3s}d} \end{pmatrix},$$

$$\Lambda_2 = \begin{pmatrix} V_{11s}\ e^{\xi_{1s}d}\ V_{12s}\ e^{\xi_{2s}d}\ V_{13s}\ e^{\xi_{3s}d} \\ V_{21s}\ e^{\xi_{1s}d}\ V_{22s}\ e^{\xi_{2s}d}\ V_{23s}\ e^{\xi_{3s}d} \\ V_{51s}\ e^{\xi_{1s}d}\ V_{52s}\ e^{\xi_{2s}d}\ V_{53s}\ e^{\xi_{3s}d} \end{pmatrix},$$

$$\Lambda_3 = \begin{pmatrix} -v_{11h}\ e^{-\xi_{1h}d}\ -v_{12h}\ e^{-\xi_{2h}d}\ -v_{13h}\ e^{-\xi_{3h}d} \\ -v_{21h}\ e^{-\xi_{1h}d}\ -v_{22h}\ e^{-\xi_{2h}d}\ -v_{23h}\ e^{-\xi_{3h}d} \\ -v_{51h}\ e^{-\xi_{1h}d}\ -v_{52h}\ e^{-\xi_{2h}d}\ -v_{53h}\ e^{-\xi_{3h}d} \end{pmatrix},$$

$$\Lambda_4 = \begin{pmatrix} v_{41s}\ e^{-\xi_{1s}d} & v_{42s}\ e^{-\xi_{2s}d} & sv_{43s}\ e^{-\xi_{3s}d} \\ K_s v_{31s}\ e^{-\xi_{1s}d} & K_s v_{32s}\ e^{-\xi_{2s}d} & K_s v_{33s}\ e^{-\xi_{3s}d} \\ -\xi_{1s}\ K_s v_{31s}\ e^{-\xi_{1s}d} & -\xi_{2s}\ K_s v_{32s}\ e^{-\xi_{2s}d} & -\xi_{3s}\ K_s v_{33s}\ e^{-\xi_{3s}d} \end{pmatrix},$$

$$\Lambda_5 = \begin{pmatrix} V_{41s}\ e^{\xi_{1s}d} & V_{42s}\ e^{\xi_{2s}d} & V_{43s}\ e^{\xi_{3s}d} \\ K_s V_{31s}\ e^{\xi_{1s}d} & K_s V_{32s}\ e^{\xi_{2s}d} & K_s V_{33s}\ e^{\xi_{3s}d} \\ \xi_{1s} K_s V_{31s}\ e^{\xi_{1s}d} & \xi_{2s} K_s V_{32s}\ e^{\xi_{2s}d} & \xi_{3s} K_s V_{33s}\ e^{\xi_{3s}d} \end{pmatrix},$$

$$\Lambda_6 = \begin{pmatrix} -v_{41h}\ e^{-\xi_{1h}d} & -v_{42h}\ e^{-\xi_{2h}d} & -v_{43h}\ e^{-\xi_{3h}d} \\ -K_0 v_{31h}\ e^{-\xi_{1h}d} & -K_0 v_{32h}\ e^{-\xi_{12h}d} & -K_0 v_{33h}\ e^{-\xi_{3h}d} \\ \xi_{1h} K_0 v_{31h}\ e^{-\xi_{1h}d} & \xi_{2h} K_0 v_{32h}\ e^{-\xi_{2h}d} & \xi_{3h} K_0 v_{33h}\ e^{-\xi_{3h}d} \end{pmatrix},$$

$$\Lambda_7 = \begin{pmatrix} v_{51s} & v_{52s} & v_{53s} \\ v_{41s} & v_{42s} & v_{43s} \\ v_{31s} & v_{32s} & v_{33s} \end{pmatrix},$$

$$\Lambda_8 = \begin{pmatrix} V_{51s} & V_{52s} & V_{53s} \\ V_{41s} & V_{42s} & V_{43s} \\ V_{31s} & V_{32s} & V_{33s} \end{pmatrix},\ \Lambda_9 = \begin{pmatrix} 0\ 0\ 0 \\ 0\ 0\ 0 \\ 0\ 0\ 0 \end{pmatrix},$$

$$\Lambda_{10} = \begin{pmatrix} u_{11s}\ e^{-\zeta_{1s}d}\ u_{12s}\ e^{-\zeta_{2s}d}\ u_{13s}\ e^{-\zeta_{3s}d} \\ u_{21s}\ e^{-\zeta_{1s}d}\ u_{22s}\ e^{-\zeta_{2s}d}\ u_{23s}\ e^{-\zeta_{3s}d} \\ u_{51s}\ e^{-\zeta_{1s}d}\ u_{52s}\ e^{-\zeta_{2s}d}\ u_{53s}\ e^{-\zeta_{3s}d} \end{pmatrix},$$

$$\Lambda_{11} = \begin{pmatrix} U_{11s}\ e^{\zeta_{1s}d}\ U_{12s}\ e^{\zeta_{2s}d}\ U_{13s}\ e^{\zeta_{3s}d} \\ U_{21s}\ e^{\zeta_{1s}d}\ U_{22s}\ e^{\zeta_{2s}d}\ U_{23s}\ e^{\zeta_{3s}d} \\ U_{51s}\ e^{\zeta_{1s}d}\ U_{52s}\ e^{\zeta_{2s}d}\ U_{53s}\ e^{\zeta_{3s}d} \end{pmatrix},$$

$$\Lambda_{12}=\begin{pmatrix} -u_{11h}\ e^{-\zeta_{1h}d} & -u_{12h}\ e^{-\zeta_{2h}d} & -u_{13h}\ e^{-\zeta_{3h}d} \\ -u_{21h}\ e^{-\zeta_{1h}d} & -u_{22h}\ e^{-\zeta_{2h}d} & -u_{23h}\ e^{-\zeta_{3h}d} \\ -u_{51h}\ e^{-\zeta_{1h}d} & -u_{52h}\ e^{-\zeta_{2h}d} & -u_{53h}\ e^{-\zeta_{3h}d} \end{pmatrix},$$

$$\Lambda_{13}=\begin{pmatrix} u_{41s}\ e^{-\zeta_{1s}d} & u_{42s}\ e^{-\zeta_{2s}d} & u_{43s}\ e^{-\zeta_{3s}d} \\ K_s u_{31s}\ e^{-\zeta_{1s}d} & K_s u_{32s}\ e^{-\zeta_{2s}d} & K_s u_{33s}\ e^{-\zeta_{3s}d} \\ -\zeta_{1s}\ K_s u_{31s}\ e^{-\zeta_{1s}d} & -\zeta_{2s}\ K_s u_{32s}\ e^{-\zeta_{2s}d} & -\zeta_{3s}\ K_s u_{33s}\ e^{-\zeta_{3s}d} \end{pmatrix},$$

$$\Lambda_{14}=\begin{pmatrix} U_{41s}\ e^{\zeta_{1s}d} & U_{42s}\ e^{\zeta_{2s}d} & U_{43s}\ e^{\zeta_{3s}d} \\ K_s U_{31s}\ e^{\zeta_{1s}d} & K_s U_{32s}\ e^{\zeta_{2s}d} & K_s U_{33s}\ e^{\zeta_{3s}d} \\ \zeta_{1s} K_s U_{31s}\ e^{\zeta_{1s}d} & \zeta_{2s} K_s U_{32s}\ e^{\zeta_{2s}d} & \zeta_{3s} K_s U_{33s}\ e^{\zeta_{3s}d} \end{pmatrix},$$

$$\Lambda_{15}=\begin{pmatrix} -u_{41h}\ e^{-\zeta_{1h}d} & -u_{42h}\ e^{-\zeta_{2h}d} & -u_{43h}\ e^{-\zeta_{3h}d} \\ -K_0 u_{31h}\ e^{-\zeta_{1h}d} & -K_0 u_{32h}\ e^{-\zeta_{2h}d} & -K_0 u_{33h}\ e^{-\zeta_{3h}d} \\ \zeta_{1h} K_0 u_{31h}\ e^{-\zeta_{1h}d} & \zeta_{2h} K_0 u_{32h}\ e^{-\zeta_{2h}d} & \zeta_{3h} K_0 u_{33h}\ e^{-\zeta_{3h}d} \end{pmatrix},$$

$$\Lambda_{16}=\begin{pmatrix} u_{51s} & u_{52s} & u_{53s} \\ u_{41s} & u_{42s} & u_{43s} \\ u_{31s} & u_{32s} & u_{33s} \end{pmatrix},$$

$$\Lambda_{17}=\begin{pmatrix} U_{51s} & U_{52s} & U_{53s} \\ U_{41s} & U_{42s} & U_{43s} \\ U_{31s} & U_{32s} & U_{33s} \end{pmatrix},\ \Lambda_{18}=\begin{pmatrix} 0 & 0 & 0 \\ 0 & 0 & 0 \\ 0 & 0 & 0 \end{pmatrix},$$

$$g_1=-\sum_{i=1}^{3}\sum_{j=1}^{3}(Q_{1,ij}v_{3is}v_{3js}\,M_{is}M_{js}\,e^{-d(\xi_{is}+\xi_{js})}+W_{1,ij}v_{3is}V_{3js}M_{is}m_{js}\,e^{y(-\xi_{is}+\xi_{js})}$$
$$+O_{1,ij}V_{3is}V_{3js}m_{is}m_{js}\,e^{d(\xi_{is}+\xi_{js})})+\sum_{i=1}^{3}\sum_{j=1}^{3}E_{1h,ij}v_{3ih}v_{3jh}M_{ih}M_{jh}\,e^{-\left(\xi_{ih}+\xi_{jh}\right)d},$$

$$g_2=-\sum_{i=1}^{3}\sum_{j=1}^{3}(Q_{2,ij}v_{3is}v_{3js}\,M_{is}M_{js}\,e^{-d(\xi_{is}+\xi_{js})}+W_{2,ij}v_{3is}V_{3js}M_{is}m_{js}\,e^{d(-\xi_{is}+\xi_{js})}$$
$$+O_{2,ij}V_{3is}V_{3js}m_{is}m_{js}\,e^{d(\xi_{is}+\xi_{js})})+\sum_{i=1}^{3}\sum_{j=1}^{3}E_{2h,ij}\ v_{3ih}v_{3jh}M_{ih}M_{jh}\,e^{-\left(\xi_{ih}+\xi_{jh}\right)d},$$

$$g_3=-\sum_{i=1}^{3}\sum_{j=1}^{3}(Q_{5,ij}v_{3is}v_{3js}\,M_{is}M_{js}\,e^{-d(\xi_{is}+\xi_{js})}+W_{5,ij}v_{3is}V_{3js}M_{is}m_{js}\,e^{d(-\xi_{is}+\xi_{js})}$$
$$+O_{5,ij}V_{3is}V_{3js}m_{is}m_{js}\,e^{d(\xi_{is}+\xi_{js})})+\sum_{i=1}^{3}\sum_{j=1}^{3}E_{5h,ij}\ v_{3ih}v_{3jh}M_{ih}M_{jh}\,e^{-\left(\xi_{ih}+\xi_{jh}\right)d},$$

$$g_4 = -\sum_{i=1}^{3}\sum_{j=1}^{3}(Q_{4,ij}v_{3is}v_{3js}\,M_{is}M_{js}\,e^{-d(\xi_{is}+\xi_{js})} + W_{4,ij}v_{3is}V_{3js}M_{is}m_{js}\,e^{d(-\xi_{is}+\xi_{js})}$$

$$+O_{4,ij}V_{3is}V_{3js}m_{is}m_{js}\,e^{d(\xi_{is}+\xi_{js})}) + \sum_{i=1}^{3}\sum_{j=1}^{3}E_{4h,ij}\;v_{3ih}v_{3jh}M_{ih}M_{jh}\,e^{-\left(\xi_{ih}+\xi_{jh}\right)d},$$

$$g_5 = -\sum_{i=1}^{3}\sum_{j=1}^{3}K_s(Q_{3,ij}v_{3is}v_{3js}\,M_{is}M_{js}\,e^{-y(\xi_{is}+\xi_{js})} + W_{3,ij}v_{3is}V_{3js}M_{is}m_{js}\,e^{d(-\xi_{is}+\xi_{js})}$$

$$+O_{3,ij}V_{3is}V_{3js}m_{is}m_{js}\,e^{d(\xi_{is}+\xi_{js})}) + \sum_{i=1}^{3}\sum_{j=1}^{3}K_0E_{3h,ij}\;v_{3ih}v_{3jh}M_{ih}M_{jh}\,e^{-\left(\xi_{ih}+\xi_{jh}\right)d},$$

$$g_6 = -\sum_{i=1}^{3}\sum_{j=1}^{3}K_s(-\left(\xi_{is}+\xi_{js}\right)Q_{3,ij}v_{3is}v_{3js}\,M_{is}M_{js}\,e^{-d(\xi_{is}+\xi_{js})}$$

$$+\left(-\xi_{is}+\xi_{js}\right)W_{3,ij}v_{3is}V_{3js}M_{is}m_{js}\,e^{d(-\xi_{is}+\xi_{js})}$$

$$+\left(\xi_{is}+\xi_{js}\right)O_{3,ij}V_{3is}V_{3js}m_{is}m_{js}\,e^{d(\xi_{is}+\xi_{js})})$$

$$+\sum_{i=1}^{3}\sum_{j=1}^{3}-\left(\xi_{ih}+\xi_{jh}\right)K_0E_{3h,ij}\;v_{3ih}v_{3jh}M_{ih}M_{jh}\,e^{-\left(\xi_{ih}+\xi_{jh}\right)d},$$

$$g_7 = -\sum_{i=1}^{3}\sum_{j=1}^{3}(Q_{5,ij}v_{3is}v_{3js}\,M_{is}M_{js}\,e^{-d(\xi_{is}+\xi_{js})}$$

$$+W_{5,ij}v_{3is}V_{3js}M_{is}m_{js}\,e^{d(-\xi_{is}+\xi_{js})} + O_{5,ij}V_{3is}V_{3js}m_{is}m_{js}\,e^{d(\xi_{is}+\xi_{js})}),$$

$$g_8 = -\sum_{i=1}^{3}\sum_{j=1}^{3}(Q_{4,ij}v_{3is}v_{3js}\,M_{is}M_{js}\,e^{-d(\xi_{is}+\xi_{js})}$$

$$+W_{4,ij}v_{3is}V_{3js}M_{is}m_{js}\,e^{d(-\xi_{is}+\xi_{js})} + O_{4,ij}V_{3is}V_{3js}m_{is}m_{js}\,e^{d(\xi_{is}+\xi_{js})}),$$

$$g_9 = -\sum_{i=1}^{3}\sum_{j=1}^{3}(Q_{3,ij}v_{3is}v_{3js}\,M_{is}M_{js}\,e^{-y(\xi_{is}+\xi_{js})}$$

$$+W_{3,ij}v_{3is}V_{3js}M_{is}m_{js}\,e^{d(-\xi_{is}+\xi_{js})} + O_{3,ij}V_{3is}V_{3js}m_{is}m_{js}\,e^{d(\xi_{is}+\xi_{js})}).$$

Appendix G

Coefficients 6

$$\begin{pmatrix} Q_{1,ij} \\ Q_{2,ij} \\ Q_{3,ij} \\ Q_{4,ij} \\ Q_{5,ij} \\ Q_{6,ij} \end{pmatrix} = \begin{pmatrix} \xi_{is}+\xi_{js} & A_{16} & 0 & 0 & A_{17} & 0 \\ A_{18} & \xi_{is}+\xi_{js} & 0 & A_{19} & 0 & 0 \\ 0 & 0 & \xi_{is}+\xi_{js} & 0 & 0 & A_{20} \\ 0 & A_{21} & 0 & \xi_{is}+\xi_{js} & A_{16} & A_{22} \\ A_{23} & 0 & A_{24} & A_{18} & \xi_{is}+\xi_{js} & 0 \\ A_{25} & 0 & A_{26} & A_{27} & 0 & \xi_{is}+\xi_{js} \end{pmatrix}^{-1} \begin{pmatrix} 0 \\ 0 \\ A_{31}\xi_{is} \\ A_{32}\xi_{is} \\ 0 \\ -A_{33} \end{pmatrix},$$

$$\begin{pmatrix} W_{1,ij} \\ W_{2,ij} \\ W_{3,ij} \\ W_{4,ij} \\ W_{5,ij} \\ W_{6,ij} \end{pmatrix} = \begin{pmatrix} \xi_{is}-\xi_{js} & A_{16} & 0 & 0 & A_{17} & 0 \\ A_{18} & \xi_{is}-\xi_{js} & 0 & A_{19} & 0 & 0 \\ 0 & 0 & \xi_{is}-\xi_{js} & 0 & 0 & A_{20} \\ 0 & A_{21} & 0 & \xi_{is}-\xi_{js} & A_{16} & A_{22} \\ A_{23} & 0 & A_{24} & A_{18} & \xi_{is}-\xi_{js} & 0 \\ A_{25} & 0 & A_{26} & A_{27} & 0 & \xi_{is}-\xi_{js} \end{pmatrix}^{-1} \begin{pmatrix} 0 \\ 0 \\ A_{31}(\xi_{is}-\xi_{js}) \\ A_{32}(\xi_{is}-\xi_{js}) \\ 0 \\ -2A_{33} \end{pmatrix},$$

$$\begin{pmatrix} O_{1,ij} \\ O_{2,ij} \\ O_{3,ij} \\ O_{4,ij} \\ O_{5,ij} \\ O_{6,ij} \end{pmatrix} = \begin{pmatrix} -(\xi_{is}+\xi_{js}) & A_{16} & 0 & 0 & A_{17} & 0 \\ A_{18} & -(\xi_{is}+\xi_{js}) & 0 & A_{19} & 0 & 0 \\ 0 & 0 & -(\xi_{is}+\xi_{js}) & 0 & 0 & A_{20} \\ 0 & A_{21} & 0 & -(\xi_{is}+\xi_{js}) & A_{16} & A_{22} \\ A_{23} & 0 & A_{24} & A_{18} & -(\xi_{is}+\xi_{js}) & 0 \\ A_{25} & 0 & A_{26} & A_{27} & 0 & -(\xi_{is}+\xi_{js}) \end{pmatrix}^{-1} \begin{pmatrix} 0 \\ 0 \\ -A_{31}\xi_{is} \\ -A_{32}\xi_{is} \\ 0 \\ -A_{33} \end{pmatrix}.$$

Appendix H

Coefficients 7

$$\sum_{n=1}^{3} v_{1ns} M_{ns} e^{-\xi_{ns}d} + \sum_{n=1}^{3} V_{1ns} m_{ns} e^{\xi_{ns}d} = \sum_{n=1}^{3} v_{1nh} M_{nh} e^{-\xi_{nh}d},$$

$$\sum_{n=1}^{3} v_{2ns} M_{ns} e^{-\xi_{ns}d} + \sum_{n=1}^{3} V_{2ns} m_{ns} e^{\xi_{ns}d} = \sum_{n=1}^{3} v_{2nh} M_{nh} e^{-\xi_{nh}d},$$

$$\sum_{n=1}^{3} v_{5ns} M_{ns} e^{-\xi_{ns}d} + \sum_{n=1}^{3} V_{5ns} m_{ns} e^{\xi_{ns}d} = \sum_{n=1}^{3} v_{5nh} M_{nh} e^{-\xi_{nh}d},$$

$$\sum_{n=1}^{3} v_{4ns} M_{ns} e^{-\xi_{ns}d} + \sum_{n=1}^{3} V_{4ns} m_{ns} e^{\xi_{ns}d} = \sum_{n=1}^{3} v_{4nh} M_{nh} e^{-\xi_{nh}d},$$

$$\sum_{n=1}^{3} K_s v_{3ns} M_{ns} e^{-\xi_{ns}d} + \sum_{n=1}^{3} K_s V_{3ns} m_{ns} e^{\xi_{ns}d} = \sum_{n=1}^{3} K_0 v_{3nh} M_{nh} e^{-\xi_{nh}d},$$

$$-\sum_{n=1}^{3} K_s \xi_{ns} v_{3ns} M_{ns} e^{-\xi_{ns}d} + \sum_{n=1}^{3} K_s \xi_{ns} V_{3ns} m_{ns} e^{\xi_{ns}d} = -\sum_{n=1}^{3} K_0 \xi_{nh} v_{3nh} M_{nh} e^{-\xi_{nh}d},$$

$$\sum_{n=1}^{3} v_{5ns} M_{ns} e^{-\xi_{ns}d} + \sum_{n=1}^{3} V_{5ns} m_{ns} e^{\xi_{ns}d} = f_1^*,$$

$$\sum_{n=1}^{3} v_{4ns} M_{ns} e^{-\xi_{ns}d} + \sum_{n=1}^{3} V_{4ns} m_{ns} e^{\xi_{ns}d} = f_2^*,$$

$$\sum_{n=1}^{3} v_{3ns} M_{ns} e^{-\xi_{ns}d} + \sum_{n=1}^{3} V_{3ns} m_{ns} e^{\xi_{ns}d} = f_3^*.$$

Appendix I

Coefficients 8

$$\sum_{i=1}^{3} u_{1is}L_{is}e^{-\zeta_{is}d}+\sum_{i=1}^{3} U_{1is}Y_{is}e^{\zeta_{is}d}+\sum_{i=1}^{3}\sum_{j=1}^{3}(Q_{1,ij}v_{3is}v_{3js}\,M_{is}M_{js}\,e^{-d(\xi_{is}+\xi_{js})}$$
$$+W_{1,ij}v_{3is}V_{3js}M_{is}m_{js}\,e^{y(-\xi_{is}+\xi_{js})}+O_{1,ij}V_{3is}V_{3js}m_{is}m_{js}\,e^{d(\xi_{is}+\xi_{js})})$$
$$=\sum_{i=1}^{3}V_{1ih}L_{ih}e^{-\zeta_{ih}d}+\sum_{i=1}^{3}\sum_{j=1}^{3}E_{1h,ij}v_{3ih}v_{3jh}M_{ih}M_{jh}\,e^{-\left(\xi_{ih}+\xi_{jh}\right)d},$$

$$\sum_{i=1}^{3} u_{2is}L_{is}e^{-\zeta_{is}d}+\sum_{i=1}^{3} U_{2is}Y_{is}e^{\zeta_{is}d}+\sum_{i=1}^{3}\sum_{j=1}^{3}(Q_{2,ij}v_{3is}v_{3js}\,M_{is}M_{js}\,e^{-d(\xi_{is}+\xi_{js})}$$
$$+W_{2,ij}v_{3is}V_{3js}M_{is}m_{js}\,e^{d(-\xi_{is}+\xi_{js})}+O_{2,ij}V_{3is}V_{3js}m_{is}m_{js}\,e^{d(\xi_{is}+\xi_{js})})$$
$$=\sum_{i=1}^{3}V_{2ih}L_{ih}e^{-\zeta_{ih}d}+\sum_{i=1}^{3}\sum_{j=1}^{3}E_{2h,ij}v_{3ih}v_{3jh}M_{ih}M_{jh}\,e^{-\left(\xi_{ih}+\xi_{jh}\right)d},$$

$$\sum_{i=1}^{3} u_{5is}L_{is}e^{-\zeta_{is}d}+\sum_{i=1}^{3} U_{5is}Y_{is}e^{\zeta_{is}d}+\sum_{i=1}^{3}\sum_{j=1}^{3}(Q_{5,ij}v_{3is}v_{3js}\,M_{is}M_{js}\,e^{-d(\xi_{is}+\xi_{js})}$$
$$+W_{5,ij}v_{3is}V_{3js}M_{is}m_{js}\,e^{d(-\xi_{is}+\xi_{js})}+O_{5,ij}V_{3is}V_{3js}m_{is}m_{js}\,e^{d(\xi_{is}+\xi_{js})})$$
$$=\sum_{i=1}^{3}V_{5ih}L_{ih}e^{-\zeta_{ih}d}+\sum_{i=1}^{3}\sum_{j=1}^{3}E_{5h,ij}v_{3ih}v_{3jh}M_{ih}M_{jh}\,e^{-\left(\xi_{ih}+\xi_{jh}\right)d},$$

$$\sum_{i=1}^{3} u_{4is}L_{is}e^{-\zeta_{is}d}+\sum_{i=1}^{3} U_{4is}Y_{is}e^{\zeta_{is}d}+\sum_{i=1}^{3}\sum_{j=1}^{3}(Q_{4,ij}v_{3is}v_{3js}\,M_{is}M_{js}\,e^{-d(\xi_{is}+\xi_{js})}$$
$$+W_{4,ij}v_{3is}V_{3js}M_{is}m_{js}\,e^{d(-\xi_{is}+\xi_{js})}+O_{4,ij}V_{3is}V_{3js}m_{is}m_{js}\,e^{d(\xi_{is}+\xi_{js})})$$
$$=\sum_{i=1}^{3}V_{4ih}L_{ih}e^{-\zeta_{ih}d}+\sum_{i=1}^{3}\sum_{j=1}^{3}E_{4h,ij}v_{3ih}v_{3jh}M_{ih}M_{jh}\,e^{-\left(\xi_{ih}+\xi_{jh}\right)d},$$

$$\sum_{i=1}^{3} K_s u_{3is}L_{is}e^{-\zeta_{is}d}+\sum_{i=1}^{3} K_s U_{3is}Y_{is}e^{\zeta_{is}yd}+\sum_{i=1}^{3}\sum_{j=1}^{3}K_s(Q_{3,ij}v_{3is}v_{3js}\,M_{is}M_{js}\,e^{-y(\xi_{is}+\xi_{js})}$$
$$+W_{3,ij}v_{3is}V_{3js}M_{is}m_{js}\,e^{d(-\xi_{is}+\xi_{js})}+O_{3,ij}V_{3is}V_{3js}m_{is}m_{js}\,e^{d(\xi_{is}+\xi_{js})})$$
$$=\sum_{i=1}^{3}K_0V_{3ih}L_{ih}e^{-\zeta_{ih}d}+\sum_{i=1}^{3}\sum_{j=1}^{3}K_0E_{3h,ij}v_{3ih}v_{3jh}M_{ih}M_{jh}\,e^{-\left(\xi_{ih}+\xi_{jh}\right)d},$$

$$\sum_{i=1}^{3} -\zeta_{is}K_s u_{3is}L_{is}e^{-\zeta_{is}d}+\sum_{i=1}^{3} \zeta_{is}K_s U_{3is}Y_{is}e^{\zeta_{is}yd}$$
$$+\sum_{i=1}^{3}\sum_{j=1}^{3}K_s(-\left(\xi_{is}+\xi_{js}\right)Q_{3,ij}v_{3is}v_{3js}\,M_{is}M_{js}\,e^{-d(\xi_{is}+\xi_{js})}$$
$$+\left(-\xi_{is}+\xi_{js}\right)W_{3,ij}v_{3is}V_{3js}M_{is}m_{js}\,e^{d(-\xi_{is}+\xi_{js})}+\left(\xi_{is}+\xi_{js}\right)O_{3,ij}V_{3is}V_{3js}m_{is}m_{js}\,e^{d(\xi_{is}+\xi_{js})})$$
$$=\sum_{i=1}^{3}-\zeta_{ih}K_0V_{3ih}L_{ih}e^{-\zeta_{ih}d}+\sum_{i=1}^{3}\sum_{j=1}^{3}-\left(\xi_{ih}+\xi_{jh}\right)K_0E_{3h,ij}v_{3ih}v_{3jh}M_{ih}M_{jh}\,e^{-\left(\xi_{ih}+\xi_{jh}\right)d},$$

$$\sum_{i=1}^{3}\ u_{5is}L_{is}e^{-\zeta_{is}d}+\sum_{i=1}^{3}\ U_{5is}Y_{is}e^{\zeta_{is}d}+\sum_{i=1}^{3}\sum_{j=1}^{3}(Q_{5,ij}v_{3is}v_{3js}\,M_{is}M_{js}\,e^{-d(\xi_{is}+\xi_{js})}$$
$$+W_{5,ij}v_{3is}V_{3js}M_{is}m_{js}\,e^{d(-\xi_{is}+\xi_{js})}+O_{5,ij}V_{3is}V_{3js}m_{is}m_{js}\,e^{d(\xi_{is}+\xi_{js})})=0,$$

$$\sum_{i=1}^{3}\ u_{4is}L_{is}e^{-\zeta_{is}d}+\sum_{i=1}^{3}\ U_{4is}Y_{is}e^{\zeta_{is}d}+\sum_{i=1}^{3}\sum_{j=1}^{3}(Q_{4,ij}v_{3is}v_{3js}\,M_{is}M_{js}\,e^{-d(\xi_{is}+\xi_{js})}$$
$$+W_{4,ij}v_{3is}V_{3js}M_{is}m_{js}\,e^{d(-\xi_{is}+\xi_{js})}+O_{4,ij}V_{3is}V_{3js}m_{is}m_{js}\,e^{d(\xi_{is}+\xi_{js})})=0,$$

$$\sum_{i=1}^{3}\ u_{3is}L_{is}e^{-\zeta_{is}d}+\sum_{i=1}^{3}\ U_{3is}Y_{is}e^{\zeta_{is}yd}+\sum_{i=1}^{3}\sum_{j=1}^{3}(Q_{3,ij}v_{3is}v_{3js}\,M_{is}M_{js}\,e^{-y(\xi_{is}+\xi_{js})}$$
$$+W_{3,ij}v_{3is}V_{3js}M_{is}m_{js}\,e^{d(-\xi_{is}+\xi_{js})}+O_{3,ij}V_{3is}V_{3js}m_{is}m_{js}\,e^{d(\xi_{is}+\xi_{js})})=0,$$
